# Histone deacetylase 9 regulates breast cancer cell proliferation and the response to histone deacetylase inhibitors

**DOI:** 10.18632/oncotarget.7564

**Published:** 2016-02-22

**Authors:** Marion Lapierre, Aurélien Linares, Mathieu Dalvai, Céline Duraffourd, Sandrine Bonnet, Abdelhay Boulahtouf, Carmen Rodriguez, Stéphan Jalaguier, Said Assou, Beatrice Orsetti, Patrick Balaguer, Thierry Maudelonde, Philippe Blache, Kerstin Bystricky, Nathalie Boulle, Vincent Cavaillès

**Affiliations:** ^1^ IRCM, Institut de Recherche en Cancérologie de Montpellier, Montpellier, France; ^2^ INSERM, U1194, Montpellier, France; ^3^ Université Montpellier, Montpellier, France; ^4^ Institut du Cancer de Montpellier, Montpellier, France; ^5^ Université de Toulouse, UPS, Laboratoire de Biologie Moléculaire Eucaryote (LBME), Toulouse, France; ^6^ CNRS, UMR5099, Toulouse, France; ^7^ Laboratoire de Biologie Cellulaire, CHU Arnaud de Villeneuve, Montpellier, France; ^8^ IRMB, Institute for Regenerative Medecine and Biotherapy, INSERM U1183, Montpellier, France

**Keywords:** breast cancer, histone deacetylase, HDAC9, HDAC inhibitors, SOX9

## Abstract

Histone lysine acetylation is an epigenetic mark regulated by histone acetyltransferases and histone deacetylases (HDAC) which plays an important role in tumorigenesis. In this study, we observed a strong overexpression of class IIa HDAC9, at the mRNA and protein levels, in the most aggressive human breast cancer cell lines (*i.e.* in basal breast cancer cells *vs* luminal ones or in malignant *vs* begnin MCF10A breast epithelial cell lines). HDAC9 overexpression was associated with higher rates of gene transcription and increased epigenetic marks on the HDAC9 promoter. Ectopic expression of HDAC9 in MCF7 luminal breast cancer cells led to an increase in cell proliferation and to a decrease in apoptosis. These effects were associated with a deregulated expression of several genes controlled by HDAC inhibitors such as *CDKN1A*, *BAX* and *TNFRSF10A*. Inversely, knock-down of HDAC9 expression in MDA-MB436 basal breast cancer cells reduced cell proliferation. Moreover, high HDAC9 expression decreased the efficacy of HDAC inhibitors to reduce cell proliferation and to regulate *CDKN1A* gene expression. Interestingly, the gene encoding the transcription factor *SOX9* was identified by a global transcriptomic approach as an HDAC9 target gene. In stably transfected MCF7 cells, *SOX9* silencing significantly decreased HDAC9 mitogenic activity. Finally, in a large panel of breast cancer biopsies, *HDAC9* expression was significantly increased in tumors of the basal subtype, correlated with *SOX9* expression and associated with poor prognosis. Altogether, these results indicate that HDAC9 is a key factor involved in mammary carcinogenesis and in the response to HDAC inhibitors.

## INTRODUCTION

Despite real improvement in patient treatment, breast cancer remains a significant global health issue and a major cause of cancer death worldwide [1]. Breast cancer is a complex and heterogeneous disease and at least five distinct molecular subtypes differing in clinical outcomes and treatment responses have been identified based on gene expression profiles [2]. Routinely, breast cancers are classified according to different parameters including stage, grade and expression of molecular targets such as estrogen, progesterone and human epidermal growth factor receptors. Adjuvant systemic treatments after surgery include chemotherapy, hormone therapy and HER2-targeted therapy [3].

A number of epigenetic aberrations have been characterized in breast cancer, including DNA methylation and various histone modifications, such as methylation, phosphorylation, ubiquitination, sumoylation and acetylation [4]. Acetylation is controlled by a balance in activity between histone acetyltransferase and histone deacetylase (HDAC). Human HDACs form a large family of 18 members classified in four groups (I to IV) based on sequence homologies [5, 6].

HDAC inhibitors (HDIs) have emerged as a promising new class of multifunctional anticancer agents [7]. These molecules can block multiple cancer related pathways and reverse epigenetic events implicated in cancer progression due in part, to their ability to enhance acetylation of a wide range of proteins, including transcription factors, molecular chaperones and structural components [8]. HDIs exert various effects in tumor cells which include cell cycle arrest, induction of apoptosis, inhibition of angiogenesis, activation or inactivation of tumor suppressor genes or oncogenes, and decreased invasion and metastases [9]. In preclinical studies, they showed synergy with radiation, chemotherapeutics or other agents such as proteasome inhibitors and have been tested in clinical trials, as single agents or in combination with other therapies [10]. Despite promising results, clinical trials with HDIs in solid tumors have not met the expected success and ongoing work aims at increasing HDI selectivity and deciphering the mechanisms underlying intrinsic resistance to these inhibitors [11]. In breast cancer, HDIs strongly modulate estrogen signaling and the response to hormone therapies [12].

In this study, we first demonstrate that class IIa HDAC9 expression is markedly increased at the transcriptional level in the most aggressive mammary tumor cell lines. Our data also indicate that deregulated expression of HDAC9 in breast cancer cells (ectopic expression in MCF7 cells and knock-down in MDA-MB436 cells) alters gene expression, cell proliferation and apoptosis. Most importantly, HDAC9 expression controls the sensitivity of breast cancer cells to HDAC inhibitors. Finally, we identified the *SOX9* gene as a new HDAC9 target gene which explained, at least partly, the effect of HDAC9 on breast cancer cell proliferation. Altogether, this work evidences an important role of HDAC9 in breast cancer cells and in their response to HDAC inhibitors.

## RESULTS

### HDAC9 is overexpressed in the most aggressive breast tumor cell lines

**Figure 1:**
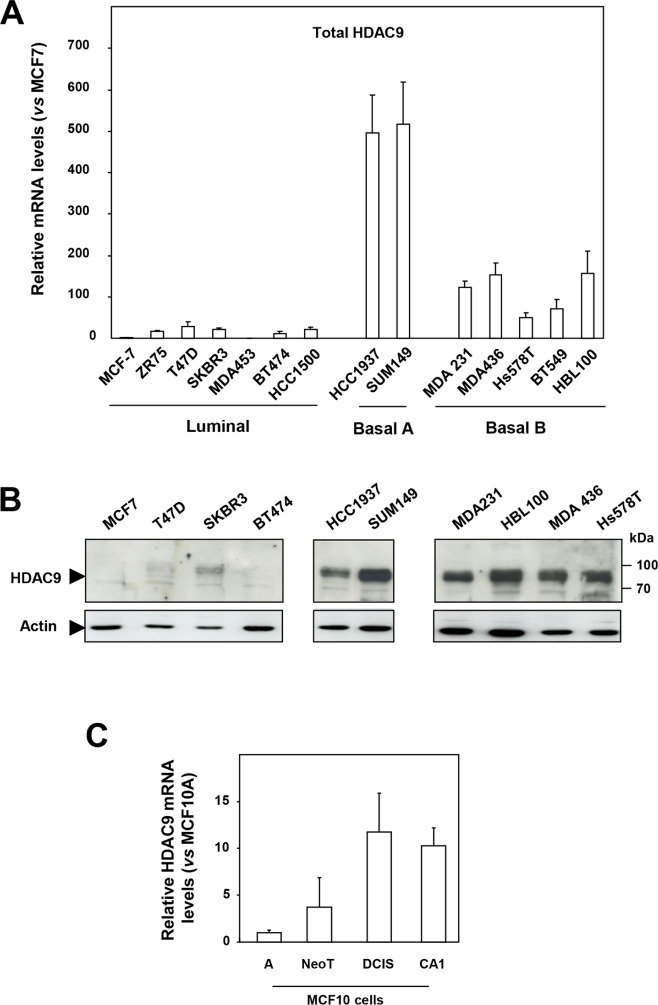
HDAC9 is overexpressed in the most aggressive breast cancer cells **A.** Total HDAC9 mRNA levels were measured in fourteen breast tumor cell lines classified as luminal (n=7), basal A (n=2) and basal B (n=5). Results are expressed relative to the HDAC mRNA levels of the MCF7 cells and represent mean ± SD of 3 independent cell cultures. **B.** Proteins were extracted from luminal (n=4), basal A (n=2) and basal B (n=4) breast tumor cells and analyzed by western-blot using anti-HDAC9 antibody. Actin was used as a loading control. This western-blot is representative of two independent experiments. **C.** Total HDAC9 mRNA levels were measured in the MCF10 mammary cell lines. Results are expressed relative to the HDAC mRNA levels of the MCF10A cells and represent mean ± SD of 3 independent cell cultures.

By comparing HDAC expression at the mRNA level in a panel of human breast tumor cell lines classified as luminal, basal A and basal B [13, 14], we found the level of HDAC9 expression to be strikingly increased in basal cells (mean ± SD = 223.7 ± 197) as compared to luminal cells (mean ± SD = 14.2 ± 10.7) (p = 0.0059) (Figure 1A). This deregulation between luminal and basal cells appeared specific since other HDACs did not display major differences in gene expression, except for HDAC4 and HDAC11, which, to a lesser extent, were respectively increased and decreased in basal cell lines (Supplementary Figure 1).

Various mRNA isoforms are encoded by the *HDAC9* gene [15]. Comparison of mRNA levels for total HDAC9 with those of the longest HDAC9 isoforms (variants 1, 4 and 5) and the MITR isoform (for *MEF2 Interacting Transcription Repressor also known as variant 3*) lacking the catalytic deacetylase domain showed a similar pattern of distribution among luminal, basal A and basal B cells (Supplementary Figure 2).

HDAC9 expression was next analyzed at the protein level by western blotting using an anti-HDAC9 antibody recognizing all HDAC9 protein isoforms (Figure 1B and Supplementary Figure 3). Low or no HDAC9 signal was detected in the luminal breast cancer cells analyzed, whereas the anti-HDAC9 antibody detected high levels of a protein around 95 kDa in the basal cell lines tested (Figure 1B and Supplementary Figure 3B). Analysis of nuclear and cytoplasmic extracts from luminal MCF7 and basal HBL100 cells showed predominant nuclear localization of the HDAC9 protein in HBL100 cells (Supplementary Figure 3C). Similar results were found in additional luminal and basal cells tested including MDA-MB436, MDA-MB231 and MDA-MB453 cells (Supplementary Figure 3D). Immunofluorescence analysis confirmed very low levels of endogenous protein in MCF7 cells and high levels of endogenous HDAC9 protein in basal cells, with a predominant and diffuse distribution in the nuclei (Supplementary Figure 4A and 4B). Altogether, these data demonstrated that HDAC9 expression was significantly increased at the mRNA and protein levels in basal breast cancer cells, which exhibit the most aggressive phenotype.

These results were confirmed using the MCF10 cell model of breast cancer progression which comprises the well-differentiated cell line MCF10A unable to form lesions in nude mice, the premalignant transformed cell line called MCF10NeoT and the two malignant subclones MCF10DCIS and MCF10CA1, which produce either comedo-type tumors or highly invasive cancers after transplantation into nude mice [16]. As compared to MCF10A cells, HDAC9 expression increased by 4 fold in MCF10NeoT cells and by 10 to 12-fold in MCF10DCIS and CA1 clones (Figure 1C) whereas the expression of the other HDACs was not significantly deregulated (Supplementary Figure 5). These data thus strengthen the conclusion that overexpression of the *HDAC9* gene might be associated with breast cancer progression.

### Mechanisms of HDAC9 overexpression in basal breast cancer cells

**Figure 2:**
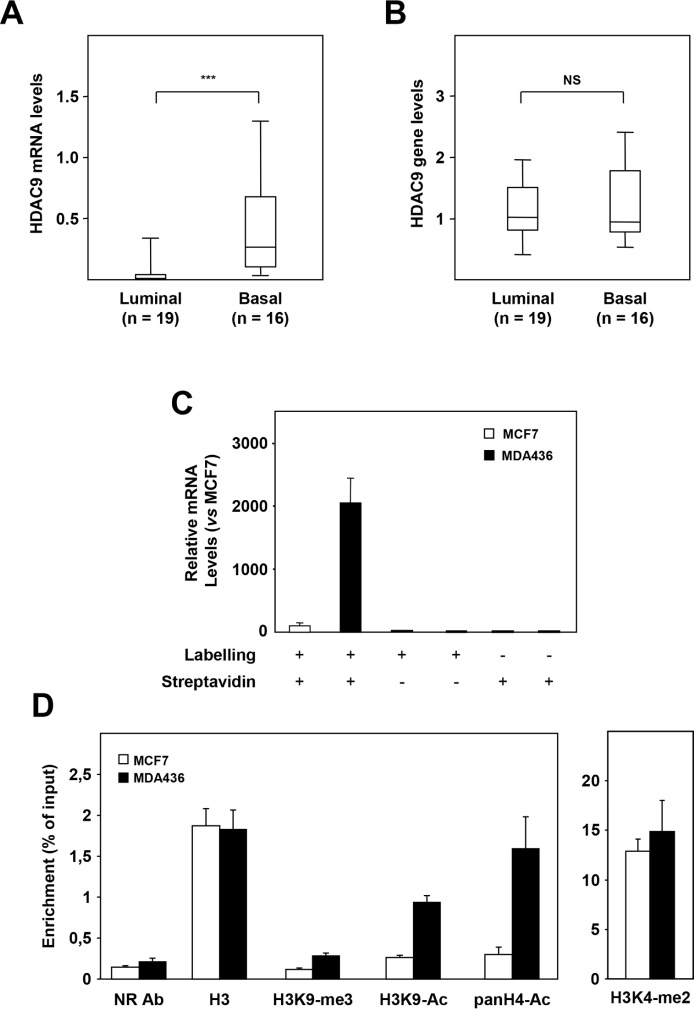
Mechanisms of HDAC9 deregulation in basal breast tumor cells **A.** HDAC9 mRNA levels were measured in luminal (n=19) and basal (n=16) breast tumor cell lines using RT-qPCR as described in Materials and Methods. **B.** Same as in panel A for HDAC9 gene levels measured by qPCR. **C.** HDAC9 transcription rates were measured in MCF7 and MDA-MB436 breast tumor cells in a run-on experiment. HDAC9 mRNA levels are expressed relative to the MCF7 cell line used as reference. The various experimental conditions used for both cell lines are indicated. **D.** ChIP experiments on the HDAC9 gene promoter after immunoprecipitation using antibodies against Histone H3 (H3), H3K9-me3, H3K9-Ac, panH4-Ac, H3K4-me2 or an irrelevant antibody (NR Ab).*** p < 0.001.

We investigated the mechanisms by which the *HDAC9* gene is overexpressed in basal breast cancer cells. In a set of 35 breast tumor cell lines classified as luminal (n=19) or basal (n=16), RT-qPCR quantification confirmed higher levels of HDAC9 mRNA levels in basal cells as compared to luminal ones (Figure 2A, p<0.0001). In the same series of cells, *HDAC9* gene amplification was analyzed by qPCR. No significant difference in *HDAC9* gene levels was found between basal and luminal cell lines suggesting that gain in *HDAC9* gene copy number is not involved in HDAC9 overexpression in basal breast cancer cells (Figure 2B). We next performed run-on experiments using luminal MCF7 and basal MDA-MB436 cell lines to compare *HDAC9* gene transcription rate in both groups of mammary tumor cells (Figure 2C). HDAC9 transcription rate was found to be significantly enhanced in MDA-MB436 cells as compared to MCF7 cells (about 20-fold), suggesting that this mechanism is crucial for the differential expression of HDAC9 between the two cell lines. To emphasize this observation, we compared several epigenetic marks on the *HDAC9* gene promoter in MCF7 and MDA-MB436 cells. As shown in Figure 2D, differences in epigenetic marks were found in the *HDAC9* gene promoter between the two cell lines, with increased levels of both H3K9 and H4 acetylation and H3K9 methylation in MDA436 as compared to MCF7 cells.

### HDAC9 increases breast cancer cell proliferation

**Figure 3:**
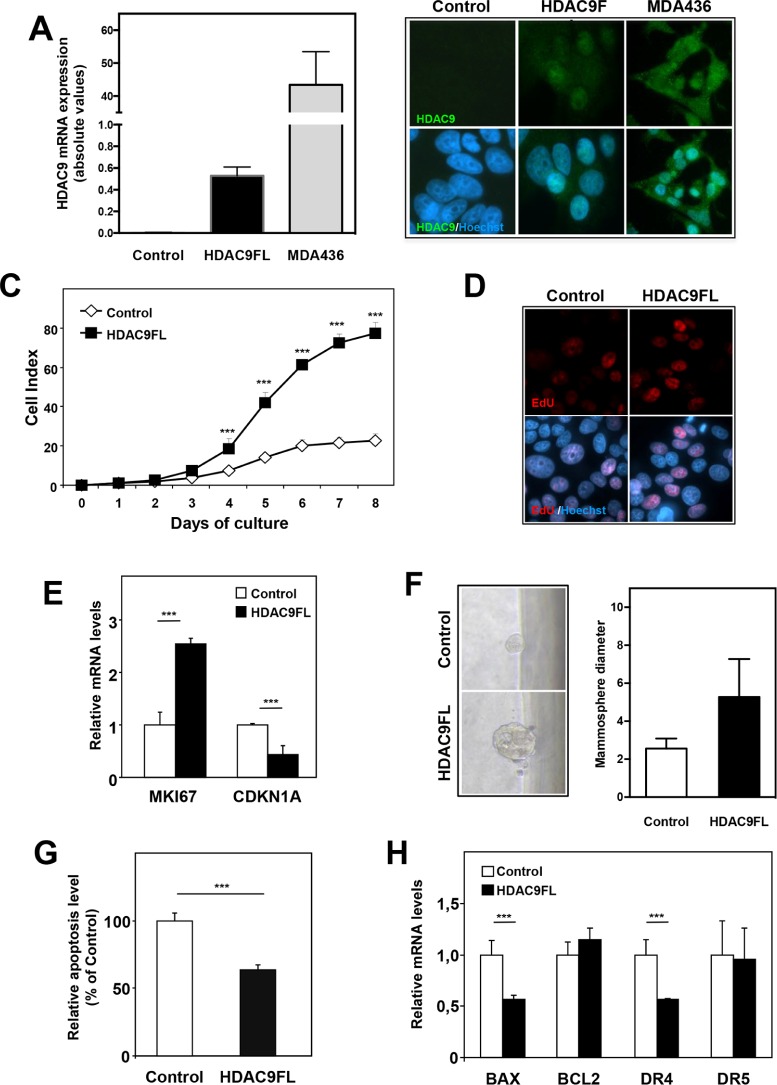
HDAC9 regulates cell proliferation and apoptosis in breast cancer cells **A.** HDAC9 mRNA levels were quantified using RT-qPCR in MCF7 cells stably transfected using either control plasmid (Control) or full length HDAC9 plasmid (HDAC9FL) and in MDA-MB436 cells. Results are expressed in arbitrary units (AU) as mean ± SD of 6 independent cell cultures. **B.** MCF7-Control, MCF7-HDAC9FL and MDA-MB436 were analyzed by immunofluorescence using anti-HDAC9 antibody and Hoechst labeling. **C.** Cell index corresponding to the number of MCF7-Control and MCF7-HDAC9FL viable cells were monitored every 24 hours during 8 days using the xCELLigence system. Values are means ± SD, n=3 independent experiments. **D.** Proliferating cells in MCF7-Control and MCF7-HDAC9FL were analyzed by immunofluorescence using EdU and Hoechst labeling. **E.** Ki67 and p21 mRNA levels were quantified using RT-qPCR. Results represent fold change ± SD of 6 independent cell cultures *vs* levels in MCF7-Control cells after normalization to 28S mRNA. **F.** Mammosphere growth was analyzed in non-adherent conditions using MCF7-Control and MCF7-HDAC9FL cells. Values represent mammosphere diameter (mean ± SD of 34 values; n=3 independent experiments). **G.** Basal apoptosis was measured using the Cell Death Detection ELISA kit. Results are expressed relative to MCF7-Control cells (100%) and represent mean ± SD of 4 wells; n=3 independent experiments. F- BAX, BCl2, DR4 and DR5 mRNA levels were quantified using RT-qPCR. Results are expressed as in D. *** p< 0.001.

As HDAC9 was found to be overexpressed in the most aggressive breast tumor cells, we wondered what consequences HDAC9 overexpression could have on luminal cells behavior. We thus stably transfected luminal MCF7 cells using a full-length HDAC9 expression vector and generated MCF7-HDAC9FL cells together with control cells transfected with the corresponding empty expression vector (MCF7-Control). Expression of HDACs from classes I, IIa, IIb and IV was not significantly altered by HDAC9 overexpression in MCF7 cells (data not shown). As shown in Figure 3A and 3B, HDAC9 mRNA levels were strongly expressed in MCF7-HDAC9FL cells although the levels appeared still very low as compared to those found in MDA-436 basal human breast cancer cells.

The effect of HDAC9 expression on cell proliferation was analyzed using the xCELLigence technology which allows real-time monitoring of cell proliferation through measurement of impedance-based signals. We found that MCF7-HDAC9FL cells exhibited a higher proliferation rate than control cells (Figure 3C). This effect was also observed by quantifying the percentage of cells incorporating EdU (Figure 3D). Moreover, MCF7-HDAC9FL cells showed higher expression of the proliferation marker *MKI67* together with decreased expression of the cell cycle inhibitor *CDKN1A* genes (Figure 3E). Interestingly, the effect on proliferation was also evidenced when cells were grown as mammospheres (Figure 3F). In an independent transfection experiment, we isolated stable MCF7 clones with two control clones (clones C3 and C4) and two HDAC9 overexpressing clones (clones 9.4 and 9.6) (Supplementary Figure 6A). The use of these stably transfected MCF7 clones further confirmed the growth advantage of HDAC9 overexpressing cells (Supplementary Figure 6B). To better characterize the effect of HDAC9, we monitored apoptosis in MCF7-HDAC9FL cells and observed that HDAC9 exerted an anti-apoptotic activity as shown by the quantification of cytoplasmic nucleosomes (Figure 3G). This apoptotic effect was in line with the significant decreased expression of two pro-apoptotic genes, *BAX* and *DR4*, in HDAC9 overexpressing cells as compared to control cells (Figure 3H).

**Figure 4:**
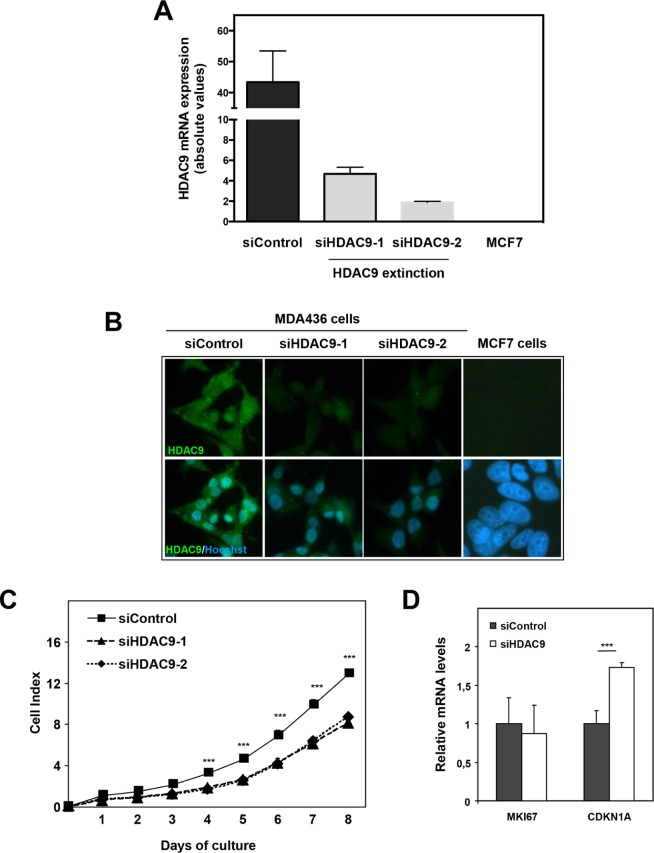
Effect of HDAC9 knock-down on breast cancer cell proliferation **A.** HDAC9 expression was measured by RT-qPCR in MDA-MB436 cells after silencing (siHDAC9-1 and siHDAC9-2) or not (siControl) of the *HDAC9* gene. Values represent fold changes ± SD corrected by the 28S mRNA and normalized to control cells. **B.** The same MDA-MB436 cells were analyzed by immunofluorescence using the anti-HDAC9 antibody. **C.** Cell index corresponding to the number of MDA436-siControl and MDA436-siHDAC9-1/-2 viable cells were monitored every 24 hours during 8 days using the xCELLigence system. Values are means ± SD, n=3 independent experiments. **D.** Ki67 and p21 mRNA levels were quantified using RT-QPCR. Results are expressed relative to the mRNA levels measured for the MDA436-siControl cells. *** p< 0.001.

The impact of HDAC9 on cell proliferation was also assessed by knocking-down its expression in basal breast cancer cells using small interfering RNA. We used two different siRNAs that decreased HDAC9 expression by more than 3-fold in MDA-MB436 basal cells as monitored at the mRNA and protein levels (Figure 4A and 4B). As shown in the Figure, the levels of HDAC9 expression after silencing in MDA-436 cells remained relatively high as compared to MCF7 cells. Moreover, HDAC9 depletion did not impact significantly the mRNA levels for the other classes of HDACs in MDA-MB436 (data not shown). In line with the data obtained in the model of HDAC9 overexpressing cells, we found that the depletion of HDAC9 expression led to a decrease in cell proliferation in MDA-MB436 cells (Figure 4C). Although, the level of *MKI67* mRNA was not affected after HDAC9 silencing, we observed a significant increase in *CDKN1A* gene expression (Figure 4D).

Finally, we also investigated whether the catalytic domain of HDAC9 was required for the regulation of cell proliferation. The ectopic expression of the MITR isoform which lacks the catalytic deacetylase domain did not increase MCF7 cell proliferation nor KI67 expression (Supplementary Figure 7). Altogether, these data demonstrate that HDAC9 is a strong regulator of breast cancer cell proliferation and survival.

### HDAC9 increases the resistance of breast cancer cells to HDIs

**Figure 5:**
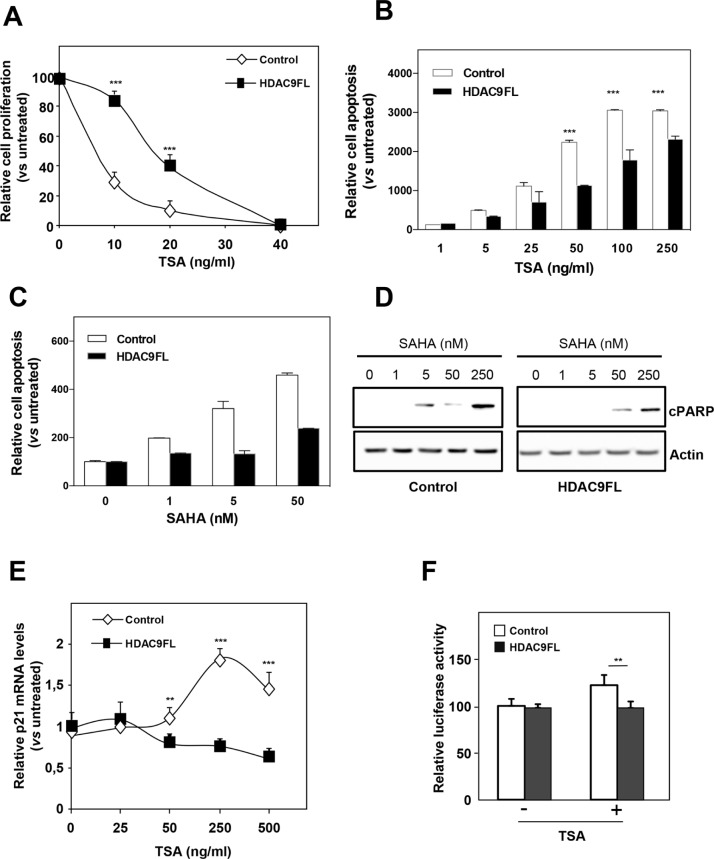
HDAC9 increases the resistance of breast cancer cells to HDIs **A.** MCF7-Control and MCF7-HDAC9FL were treated with increasing concentrations of TSA (up to 40 ng/ml) or with solvent alone (Control) and viable cells were monitored using the xCELLigence system during 72 hours. Values are means ± SD, n=3 independent experiments. **B.** MCF7-Control and MCF7-HDAC9FL were treated with increasing concentrations of TSA (up to 250 ng/ml) or with solvent alone (Control) and apoptosis was measured using the Cell Death Detection ELISA kit. Results are expressed as arbitrary units, normalized to Control and represent mean ± SD of 4 wells; n=3 independent experiments. **C.** Same as in panel B with SAHA treatment. **D.** Total proteins were extracted from MCF7-Control and MCF7-HDAC9FL cells treated with increasing concentrations of SAHA (up to 250nM) and analyzed by western-blot using anti-cleaved PARP antibody. Actin was used as a loading control. **E.** Total RNA was extracted from stably transfected MCF7 cells treated or not by TSA and p21 mRNA levels were quantified using RT-qPCR. Results represent mean and SD of 3 independent cell cultures and are expressed relative to the p21 mRNA levels of the not treated cells, used as reference. **F.** MCF7-Control and MCF7-HDAC9FL were transfected with the p21 promoter luciferase reporter plasmid and treated or not by TSA (ng/ml). Results represent the luciferase activity measured after normalization for renilla luciferase activity and relative to the values obtained in the untreated MCF7-Control. Data represent mean ± SD of triplicates and are representative of 2 independent experiments. ** p< 0.01; ***p< 0.001.

We previously reported that breast cancer cells of basal type were more resistant to the antiproliferative effects of HDIs than luminal cells ([17] and Supplementary Figure 8A for a comparison of MCF7 and MDA-MB436 cells). In order to precise the role of HDAC9 expression in the response to HDIs, we analyzed the effect of trichostatin A (TSA), a pan-HDAC inhibitor, on the proliferation of MCF7-Control and MCF7-HDAC9FL cells (Figure 5A). Very interestingly, we found that MCF7-HDAC9FL cells were more resistant to TSA than control MCF7 cells (IC_50_ of 22,8 and 4,9 ng/ml, respectively). The same difference in apoptosis induction was observed in response to SAHA (Supplementary Figure 8B). The same difference in sensitivity was observed on the stably transfected MCF7 clone 9.6 in response to TSA or to the LBH589 compound, another pan-HDAC inhibitor (Supplementary Figure 8C and 8D).

Moreover, a different response to HDIs was also observed on cell apoptosis in response to TSA or SAHA as shown by quantification of the release of nucleosomes in the cytoplasm (Figure 5B and 5C). This was confirmed by measuring the expression of cleaved PARP in response to SAHA treatment (Figure 5D). The inverse results were obtained for the response to SAHA in MDA-MB436 cells after HDAC9 silencing (Supplementary Figure 9).

We also analyzed the response to HDIs at the level of gene expression regulation. In our previous work, we noticed that the regulation of *CDKN1A* expression was more sensitive to HDIs in luminal cells than in basal cells [17]. In line with this observation, we observed that TSA regulation of *CDKN1A* gene expression (endogenous mRNA levels or transiently transfected *CDKN1A* luciferase reporter vector) was diminished or abolished in MCF7-HDAC9FL cells (Figures 5E and 5F). These data thus suggest that HDAC9 expression is a key determinant of the response of breast cancer cells to HDI treatment.

### SOX9 is a target gene of HDAC9

To identify HDAC9 target genes in breast cancer cells, we performed a global transcriptome analysis of MCF7-Control and MCF7-HDAC9FL cells. We identified 315 statistically significant differentially expressed genes with a fold change (FC) >2 and a False Discovery Rate (FDR) ≤0.05, with 195 genes up-regulated in MCF7-HDAC9FL and 120 genes down-regulated (see Supplementary Tables 1 and 2 for the list of up- and down-regulated genes). The molecular signatures of MCF7-Control and MCF7-HDAC9FL cells were visualized by hierarchical clustering on the 315 genes (Supplementary Figure 10). As showed in dendrogram, all the samples of the MCF7-HDAC9FL cells self-cluster into one branch and all samples of MCF7-Control samples self-cluster into another branch. Interestingly, the GO biological process enrichment analysis of these transcriptomic data using the Ingenuity software revealed that the most significantly deregulated processes were cell death and survival, cell movement and cell growth and proliferation (see the list of the corresponding genes in Supplementary Table 3), thus supporting our *in vitro* observations.

**Figure 6:**
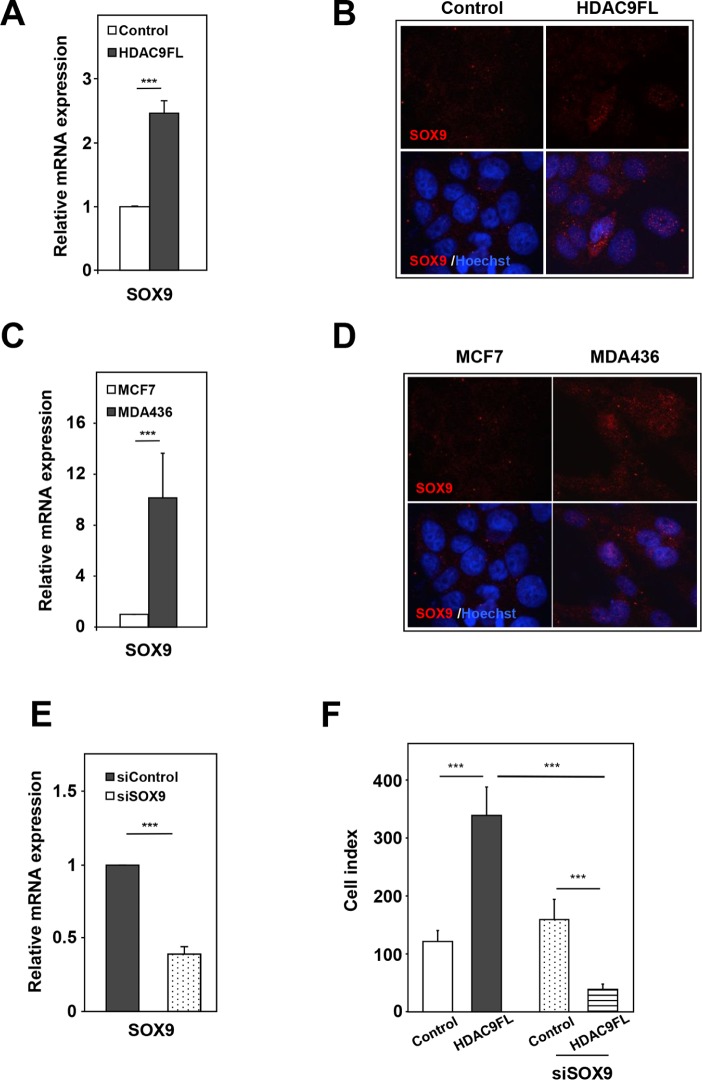
SOX9 is a target gene of HDAC9 in breast cancer cells **A.** SOX9 mRNA levels were quantified in MCF7-Control (Control) and MCF7-HDAC9FL (HDA9FL) cells using RT-qPCR. Results represent fold change ± SD of 6 independent cell cultures *vs* levels in MCF7-Control cells after normalization to 28S mRNA. **B.** Results were confirmed by immunofluorescence analysis using anti-SOX9 antibody. **C** and **D.** Same as in panel A and B, respectively in MCF7 and MDA-MB436 wild-type cells. **E.** SOX9 mRNA levels was measured by RT-qPCR in MCF7-Control cells after silencing (siSOX9) or not (siControl) of the *SOX9* gene. Results are expressed relative to the SOX9 mRNA levels of the MCF7-Control cells and represent mean ± SD of 3 independent cell cultures. **F.** MCF7-Control and MCF7-HDAC9FL viable cells, after silencing or not of the *SOX9* gene, were monitoring using the XCELLigence system during 72 hours. Values are normalized to the MCF7-Control cells and are means ± SD, n=3 independent experiments.

One of the most significantly upregulated gene was *SOX9* [sex-determining region Y (SRY)-box 9 protein], a member of the SOX family of transcription factors which exhibits an high mobility group (HMG) box DNA-binding domain and plays key roles in cell fate specification, stem cell biology and related human diseases including cancer [18]. As expected from transcriptomic data, SOX9 expression was increased at the mRNA and protein levels by 2- to 3-fold in MCF7-HDAC9FL cells as compared to MCF7-Control cells (Figure 6A and 6B). The effect of HDAC9 on *SOX9* gene expression was further confirmed by the decreased levels of SOX9 mRNA detected in HDAC9-depleted MDA-MB436 basal cells (Supplementary Figure 11A). Interestingly, similar to HDAC9, endogenous SOX9 mRNA and protein levels were significantly increased in MDA-MB436 cells as compared to MCF7-Control cells (Figure 6C and 6D). Moreover, *SOX9* gene expression paralleled that of the *HDAC9* gene in the MCF10 cells progression model except for MCF10CA1 cells in which overexpression of SOX9 was lower than in MCF10 DCIS (Supplementary Figure 11B).

A recent study reported that SOX9 plays a critical role in supporting mammary epithelial stem cells and enhances breast cancer cell proliferation and metastasis [19, 20]. In order to define whether SOX9 mediated the mitogenic effect of HDAC9 in breast cancer cells, we modulated *SOX9* gene expression in MCF7 cells overexpressing or not HDAC9 (Figure 6E). As shown in Figure 6F, we found that HDAC9 no longer increased cell proliferation but rather decreased it when the expression of *SOX9* gene was knocked-down. Altogether these results demonstrate that the *SOX9* gene is an HDAC9 target gene, which controls its mitogenic effect in breast cancer cells.

### HDAC9 expression in human breast cancer biopsies

To validate our *in vitro* data and to characterize HDAC9 expression in tumors from patients, we first reanalyzed a cDNA array data set containing 184 breast cancers mRNA profiles (NKI dataset) [21]. This analysis confirmed the data obtained on cell lines with a significant differential expression of the HDAC9 gene (p<0.0002) when comparing tumors of luminal (141 patients) and basal (43 patients) phenotypes (Table 1A). To emphasize these results, we reanalyzed another public data set (GSE2250) which confirmed the increased expression of HDAC9 (p<0.0085) in tumors of the basal subtype (56±13 *vs* 67±14 arbitrary units) [22]. In the same datasets, the expression of the *SOX9* gene was significantly increased in tumors of basal phenotype (p<0.00001 and p<0.001, respectively). As expected, and as shown in Table 1B, SOX9 expression was found to be significantly higher in tumors expressing high levels of HDAC9, both in the NKI cohort (p<0.009) and in the 189 samples of the GSE2990 dataset (p<0.0029) [23]. In the later cohort, Pearson's analysis confirmed a strong correlation between HDAC9 and SOX9 expression in breast tumors (r=0.526, p<0.00001).

**Table 1:** Expression of HDAC9 and SOX9 in human breast cancer samples

A				
Dataset	Subtype	n	*HDAC9*	*SOX9*
NKI [21]	Luminal	141	p<0,0002	p<0,00001
	Basal	43		
GDS2250 [22]	Non-basal	20	p<0,0085	p<0,001
	Basal	18		

B			
Dataset	HDAC9 groups	n	*p value*
NKI [21]	Low	119	<0,009
	High	119	
GSE2990 [23]	Low	94	<0,0029
	High	95	

C						
Dataset	Reference	n	No. of any event	p-value	Hasard Ratio (HR)	95% confidence interval (CI)
GSE25055	Hatzis et al., 2011	309	65	0.0001	1.72	1.31 - 2.28
GSE26971	Filipits et al., 2011	258	58	0.0015	1.53	1.18 - 1.99
GSE3143	Bild et al., 2006	158	50	0.0176	1.41	1.06 - 1.87
GSE33926	Kuo et al., 2012	51	12	0.0329	2.24	1.07 - 4.68
GSE22219	Buffa *et al.*, 2011	82	33	0.0447	1.78	1.01 - 3.14

Finally, to determine whether HDAC9 expression was associated with patient survival, we used the Breast Cancer Gene-Expression Miner v3 statistical mining module [24]. As shown in Table 1C, the analysis of several datasets including GSE25055 [25], GSE26971 [26], GSE3143 [27], GSE33926 [28] and GSE22219 [29], indicated that high levels of HDAC9 mRNA were significantly associated with poor prognosis (p-value ranging from 0.0001 to 0.044 with hazard ratios ranging from 1.41 to 2.24). Altogether these data strengthened our *in vitro* results concerning the correlation between HDAC9 and SOX9, their higher expression in basal type breast cancers and the deleterious effect of HDAC9 overexpression on breast cancer cell proliferation.

## DISCUSSION

Previous studies have underlined the potential role of HDACs in breast tumor progression [for a review, see [12]]. In the present study, we performed a careful analysis of HDAC expression in breast cancer cell lines. Our results demonstrate that class IIa HDAC9 may have a specific role in breast carcinoma as it is overexpressed in the most aggressive breast tumor cell lines and tumors, stimulates cell proliferation and increases resistance to HDAC inhibitors.

Since its cloning [30], HDAC9 has been implicated in various physiological processes, particularly through the analysis of HDAC9 knock-out mice [31, 32, 33, 34]. Fewer data have been published on HDAC9 in the context of cancer. HDAC9 has been shown for instance to bind and deacetylate TRIM29 and to decrease its cell proliferation-promoting activity [36]. To our knowledge, very few data on HDAC9 expression in breast tissues have been published to date, and the results presented herein evidence for the first time a strong deregulation of HDAC9 expression in breast cancer cells. We showed that HDAC9 overexpression was associated to cells of the most aggressive basal subtypes as described by gene-expression models [13, 14]. According to these models, the luminal signature includes many genes associated with the estrogen signaling pathway. By reanalyzing previous data from cDNA arrays, we observed a significant increase in HDAC9 mRNA levels in basal like breast tumors as compared to luminal ones and in ERα negative tumors as compared to ERα expressing ones (Linares *et al*, in preparation), suggesting the biological relevance of the results obtained using breast cancer cell lines. The low expression of *HDAC9* gene in breast luminal cells or tumors is in line with the recent observation that HDAC9 negatively regulates the expression of ERα in mouse cardiac tissue [37]. Interestingly, increased expression of HDAC9 was also observed in the MCF10 cell model of breast tumor progression.

HDAC9 overexpression was mainly explained by higher rates of *HDAC9* gene transcription as shown by run on experiments. Accordingly, analysis of mRNA levels for the longer HDAC9 mRNA isoforms (variants 1, 4 and 5) and the deleted HDAC9ΔCD isoform (variant 3) showed similar profiles in various breast cancer cells, suggesting that the deregulation of HDAC9 expression mainly occurs at the transcriptional level. The molecular mechanisms leading to enhanced *HDAC9* gene transcription in basal cells remain to be determined. Different epigenetic marks were detected between luminal MCF7 and basal MDA-MB436 cells on the *HDAC9* gene promoter. Alternatively, differences in transcription factors and/or cofactors may be involved in *HDAC9* gene transcription between the two groups of breast tumor cells. Among them, the MEF2 family of transcription factors is a major candidate to study as the *HDAC9* gene was shown to be a direct transcriptional target of MEF2 *in vitro* and *in vivo* [38].

Our data demonstrated that the overexpression of HDAC9 in the most aggressive breast cancer cells influences cell homeostasis with an increase in cell proliferation and a decrease in programmed cell death. This effect may be linked in part to the regulation of expression of key genes by HDAC9 such as the cell cycle inhibitor *CDKN1A* and the pro-apoptotic genes, *BAX* and *DR4.* Interestingly, our data indicated that the effect on cell proliferation was observed when cells were grown as mammospheres and further work will be necessary to define whether HDAC9 expression is deregulated in breast cancer stem cells [39]. Moreover, our preliminary experiments suggested that HDAC9 knock-down in MDA-436 cells reduced cell migration ability (data not shown) suggesting that HDAC9 might regulate the epithelial-mesenchymal transition. Interestingly, all these data are supported by our data from the global transcriptome analysis which revealed that proliferation and invasion are amongst the biological processes that are regulated by HDAC9 at the gene expression level.

The effect of HDAC9 on cell proliferation is coupled to a reduced sensitivity of breast cancer cells to the antiproliferative and apoptotic activity of HDIs, similar to that observed in cells of basal type as compared to luminal ones. Once again, this decreased sensitivity to HDIs was linked to a weaker transcriptional effect of these compounds on *CDKN1A* gene, suggesting that the *CDKN1A* gene may be a crucial target gene mediating HDAC9 effects. Based on our data, HDAC9 level may be considered as a predictive marker of the cellular response to HDAC inhibition. Other genes such as HR23B [40, 41] have been identified as biomarkers for tumor sensitivity to HDI-based therapy and the putative link of these genes with HDAC9 expression remains to be elucidated.

Finally, we identified *SOX9* as an HDAC9 target gene in breast cancer cells which supports, at least partly, its mitogenic activity. Previous work has shown that SOX9, in cooperation with Slug, supports mammary epithelial stem cells and enhances breast cancer cell metastasis [19]. Recent studies showed that SOX9 was highly expressed in breast tumor cells and tissues of basal type [20] and that patients with higher SOX9 mRNA level had significantly shorter overall survival [42], thus supporting our data. The molecular pathways by which SOX9 mediates the mitogenic effects of HDAC9 in breast tumors are not known; it may involve the Wnt/β-catenin pathway as shown by Wang et al. [20]. In this latter study, Wnt was found to increase SOX9 gene expression. We show here that class IIa HDAC9 may be another regulator of SOX9 expression in breast tumor cells.

No link between SOX9 and HDACs has been clearly reported until now. To our knowledge, a single study described that HDIs regulates the translocation of SOX9 to the nucleus and increases SOX9 acetylation [43] suggesting that SOX9 activity is regulated both at the transcriptional and post-transcriptional levels, including *via* HDACs and HAT activity. It will be necessary to undertake further work in order to decipher the molecular mechanisms underlying the deregulated expression of the *SOX9* gene in breast cancer.

In conclusion, our data show for the first time that class IIa HDAC9 could be involved at different levels in breast carcinogenesis. These data thus support previous results obtained in medulloblastoma showing that HDAC9 increased cell growth and viability and could be considered as an independent risk factor (high expression being significantly associated with poor overall survival) [35]. Obviously, other HDACs could be also highly relevant in breast cancer. For instance HDAC4 whose expression seems to be higher in basal breast cells (see Supplementary Figure 1) has also been shown to stimulate MCF7 breast cancer cell proliferation [44]. The prognostic significance of HDAC9 expression in breast tumors and its relation to the resistance of the tumor to treatment will be important issues. This might help to better define the role of HDACs in breast tumorigenesis and to propose new therapies, including HDAC inhibitors, particularly in the context of hormonal resistance.

## MATERIALS AND METHODS

### Plasmids and reagents

FLAG-tagged full length HDAC9 (HDAC9FL) plasmid was a kind gift from Dr A. Zelent [15]. For stable transfections, pcDNA3.1-HDAC9-puromycin plasmid was obtained by subcloning HDAC9FL into the pcDNA3.1 vector (Invitrogen, Cergy Pontoise, France) conferring puromycin resistance. The PWWP reporter construct containing the promoter of the *CDKN1A* gene was previously described [17]. The pRL-CMV (Promega, Charbonnières, France) was used for normalization. Zeocin was purchased from InvivoGen (Toulouse, France). HDAC inhibitors TSA and LBH 589 were purchased from Sigma Aldrich and Alinda Chemicals Ltd respectively.

### Cell lines and culture

The 14 breast cancer cell lines used in this study were obtained from the American Type Culture Collection (http://www.atcc.org/) except for the HCC1500 cell line, which was kindly provided by Dr E. Charafe-Jauffret (Marseille, France). Cells, classified as luminal (MCF7, ZR75, T47D, SKBR3, MDA-MB453, BT474, HCC1500), basal A (HCC1937, SUM149) and basal B (MDA-MB231, MDA-MB436, Hs578T, BT549, HBL100), were grown using the recommended culture conditions [13, 14]. The second set of 35 breast cancer cell lines used for *HDAC9* gene quantification has been described previously [45]. The MCF7 and MDA-MB436 cells have been authenticated using the STR typing assay (Eurofins Genomics, Germany).

MCF7 human breast adenocarcinoma cells were stably transfected with the pcDNA3.1 and pcDNA3.1-HDAC9-puromycin plasmids respectively. Pools of puromycin resistant cells (respectively named MCF7-Control and MCF7-HDAC9FL) were selected and grown in DMEM/F12 medium supplemented with 10% FCS, 100U/ml penicillin, 100mg/ml streptomycin, 100mg/ml sodium pyruvate and 0.5μg/ml puromycin. MCF7-Control and MCF7-HDAC9FL cells were also infected by lentivirus allowing overexpression of SOX9 or transiently transfected with small interfering RNA against SOX9 (Dharmacon). MDA-MB436 cells were transiently transfected with either mock siRNA (SiControl) or two sets of siRNA directed against HDAC9 gene (siHDA9-1 and siHDAC9-2) (Dharmacon). All transfections were carried out using Lipofectamine 2000 (Life Technologies) as recommended by the manufacturer.

An independent series of stable transfection was also set up using expression vectors bearing the resistance to zeomycin. The pcDNA3.1-HDAC9-zeomycin plasmid was obtained by subcloning HDAC9FL into the pcDNA3.1zeo vector (Life Technologies). In these experiments, independent clones (Clone 9.4 and 9.6) were isolated by selection through resistance to zeocin (0.5% in DMEM supplemented with 10% FCS).

For mammosphere formation, cells were plated at a density of 1 cell per well in 96-well tissue culture plates covered with poly-2-hydroxyethyl-methacrylate (Sigma) to prevent cell attachment. The medium was serum-free DMEM supplemented with 1% L-glutamine, 1% penicillin/streptomycin, 30% F12 (Sigma), 2% B27 (Invitrogen), 20 ng/ml EGF (Sigma) and 20 ng/ml bFGF (Invitrogen).

### Breast tumor samples

The breast tumor data set (NKI set) used for HDAC9 and Sox9 mRNA analysis was obtained from a cDNA array study previously described [21]. We also reanalyzed two expression datasets (GDS2250 and GSE2990) obtained from Geoprofile. The Breast Cancer Gene-Expression Miner v3 statistical mining module (targeted prognostic analysis) [24] was used to determine the correlation with survival on five different cohorts (GSE25055, GSE26971, GSE3143, GSE33926 and GSE22219). The HDAC9 gene expression values were dichotomised according to gene median.

### Luciferase reporter assays

MCF7-Control and MCF7-HDAC9FL cells were plated in 96-well plates (3.10^4^ cells per well) 24h prior to transfection with the *CDKN1A* gene promoter reporter vector using Jet-PEI (200 ng of total DNA) and treated with TSA during 24h. The pRLCMVBis plasmid (Ozyme) was used to normalize transfection efficiency. Firefly luciferase values were measured and normalized by the Renilla luciferase activity.

### Cell proliferation measurement

Stably transfected MCF7 cells and MDA-MB436 cells were seeded at a density of 2500 cells/well into E-Plate 16 (ACEA Biosciences, Inc., San Diego, CA) containing 150μl per well of medium supplemented with 10% FCS. Dynamic monitoring of cell growth was determined every 24 hours up to 8 days using the impedance-based xCELLigence system (ACEA Biosciences). The cell index was derived from measured cell-electrode impedance that correlates with the number of viable cells.

### Cell death analysis

Cells were plated in 6-well plates (50 000 cells/well) and treated or not with TSA during 24h. Apoptosis was quantified using the Cell Death Detection ELISA assay (Roche Molecular Biochemicals), according to the manufacturer's conditions. Values from absorbance measurements at 405 nm were corrected using DNA quantification in separate wells treated in parallel.

### Western-blot analysis

Whole-cell extracts, nuclear and cytoplasmic protein extracts were prepared using the NE-PER kit (Thermo Scientific) and western blotting were analyzed as previously described, using 30 μg protein for analysis [46] and a specific primary antibody against HDAC9 (abcam18970) or actin (Sigma). Signals were revealed using a horseradish peroxidase-conjugated secondary antibody (Jackson ImmunoResearch) and enhanced chemiluminescence (ECL-Plus; GE Healthcare) according to the manufacturer's instructions.

### Immunofluorescence analysis

Breast cancer cells were fixed in 4% formaldehyde, permeabilized with 1% Triton X-100 and incubated with 1% bovine serum albumin for 3h to reduce non-specific binding at room temperature. The cells were then incubated with antibodies specific for HDAC9 (Abcam ab18970) or SOX9 (Abcam ab3697). Detection was performed using an Alexa-conjugated secondary antibody (Life Technologies). After washing, sections were counterstained with Hoechst (Sigma Aldrich) and mounted for fluorescence microscopy. Negative controls using rabbit or mouse IgGs were performed and no staining was observed in these conditions.

### Real-time quantitative PCR

Total RNA was extracted from cells using High Pure RNA Isolation kit (Roche Applied Science) according to the manufacturer's instructions. Total RNA (1μg) was subjected to reverse-transcription using Superscript II reverse transcriptase (Invitrogen). RT-qPCR were performed with the LightCycler^®^ 480 SYBR Green I Master (Roche Applied Science) Melting curves of the PCR products were analyzed using the LightCycler^®^ software to exclude amplification of unspecific products. Results were normalized to 28S and TBP housekeeping gene transcripts. The primers for HDAC1 to HDAC11, p21, BAX, BCL2, DR4, DR5 and TBP genes have been described elsewhere [46]. Other primers used in this study are depicted in Supplementary Table 4.

### ChIP analysis

For ChIP analysis, MCF7 and MDA-MB436 cells (70% confluent) were cross-linked with 3,7% formaldehyde during 10 min at 37°C. The Champion ChIP One-Day Kit (Qiagen) was then used according to the manufacturer's recommendations. Immunoprecipitations were performed using rabbit polyclonal antibodies against acylated-H3 (06-599, Upstate), or an irrelevant IgG antibody as a control. Quantitative PCR was then performed using a LightCycler^®^ 480 SYBR Green I Master (Roche Applied Science) with 2μl of material per point. Primers within the HDAC9 promoter are given in Supplementary Table 4. The input DNA fraction corresponded to 5% of the amount of immunoprecipitated chromatin.

### Nuclear run-on assay

Nuclear run-on assays were performed using 6.10^7^ MCF7 or MDA-MB436 cells and biotin-streptavidin labeling as previously described [47]. RNA was purified using Trizol reagent (Invitrogen). Reverse transcription and qPCR reactions were carried out as already described.

### Global gene expression analysis

Global transcriptome analysis of MCF7-Control and MCF7-HDAC9FL cells was performed using human oligonucleotide HG-U219 microarrays (Affymetrix GeneAtlas) processed in the Microarray Core Facility of the Institute for Regenerative Medecine and Biotherapy (IRMB), CHRU-INSERM-UM Montpellier (http://www.chu-montpellier.fr/fr/irmb/). After image processing with the Affymetrix GeneChip command consol, the CEL files were analyzed using the Affymetrix Expression Console™ Software v1.3.1 and normalized with the RMA algorithm. Gene annotation was performed using NetAffx (http://www.affymetrix.com; October 2014). The Significant Analysis of Microarrays (SAM) software with the Wilcoxon test and sample label permutation (300) was used to identify differentially expressed genes between the MCF7-Control and MCF7-HDAC9FL samples. We identified 315 statistically significant differentially expressed genes with a fold change (FC) >2 and a False Discovery Rate (FDR) ≤0.05, with 195 genes up-regulated in MCF7-HDAC9FL and 120 genes down-regulated. Hierarchical clustering analyses based on the expression levels of the differentially expressed genes were performed by using the CLUSTER and TREEVIEW software packages [48]. The gene ontology (GO) biological process enrichment analyses of the differentially expressed genes were generated by Ingenuity Pathway Analysis (IPA) software. The 315 genes were imported into IPA database and were categorized on the basis of their biological process and molecular functions by using the software (www.ingenuity.com).

### Statistical analysis

Results are expressed as mean ± standard deviations (SD). Statistical analysis was performed using Student's t test or Mann-Whitney U test for comparison of two groups. A probability level of 0.05 was chosen for statistical significance. Statistical analysis was performed using GraphPad Prism 5 version 5.01 (GraphPad Software, San Diego, CA, USA). The Mann-Whitney test (StatEL software) was used for statistical analysis of the GEO profile datasets. For the Breast Cancer Gene-Expression Miner analysis [24], p-values were determined using the Cox regression model.

## SUPPLEMENTARY FIGURES AND TABLES




