# Supplementary material for: Histone deacetylase 9 regulates breast cancer cell proliferation and the response to histone deacetylase inhibitors
Source: Oncotarget. 2016 Feb 22;7(15):19693–708. doi: 10.18632/oncotarget.7564 (PMC4991412; doi:10.18632/oncotarget.7564)
Supplement: Supplementary file 1 [file oncotarget-07-19693-s001.pdf]

SUPPLEMENTARY FIGURES AND TABLES

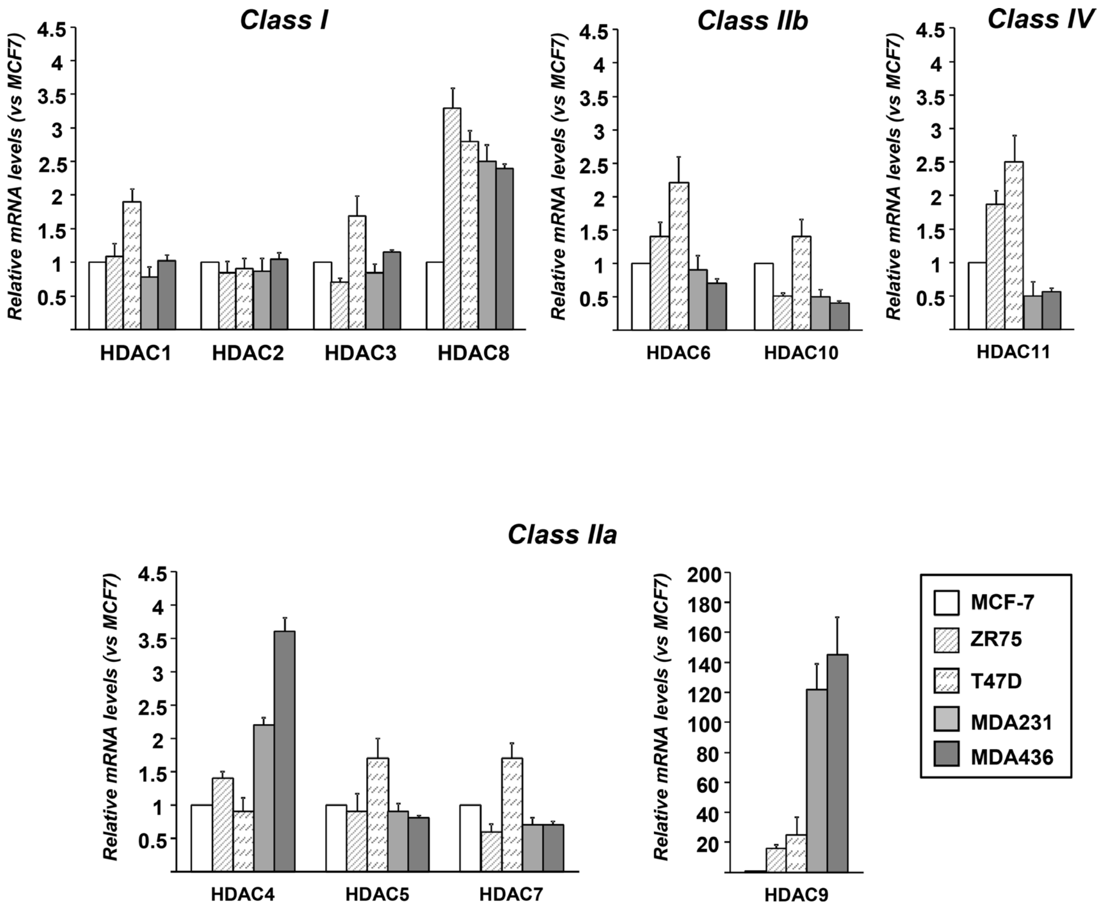

Supplementary Figure S1: HDAC expression in breast cancer cell lines. Breast tumor cell lines of luminal (MCF7, ZR75, T47D) or basal (MDA-MB231, MDA-MB436) type were grown at confluence in FCS containing medium. The mRNA levels for class I, II and IV HDACs were measured using RT-qPCR. Results are expressed relative to the MCF7 cell line used as reference. Note that a different scale was used for HDAC9. Results represent mean ± S.D. of 3 independent cell cultures.

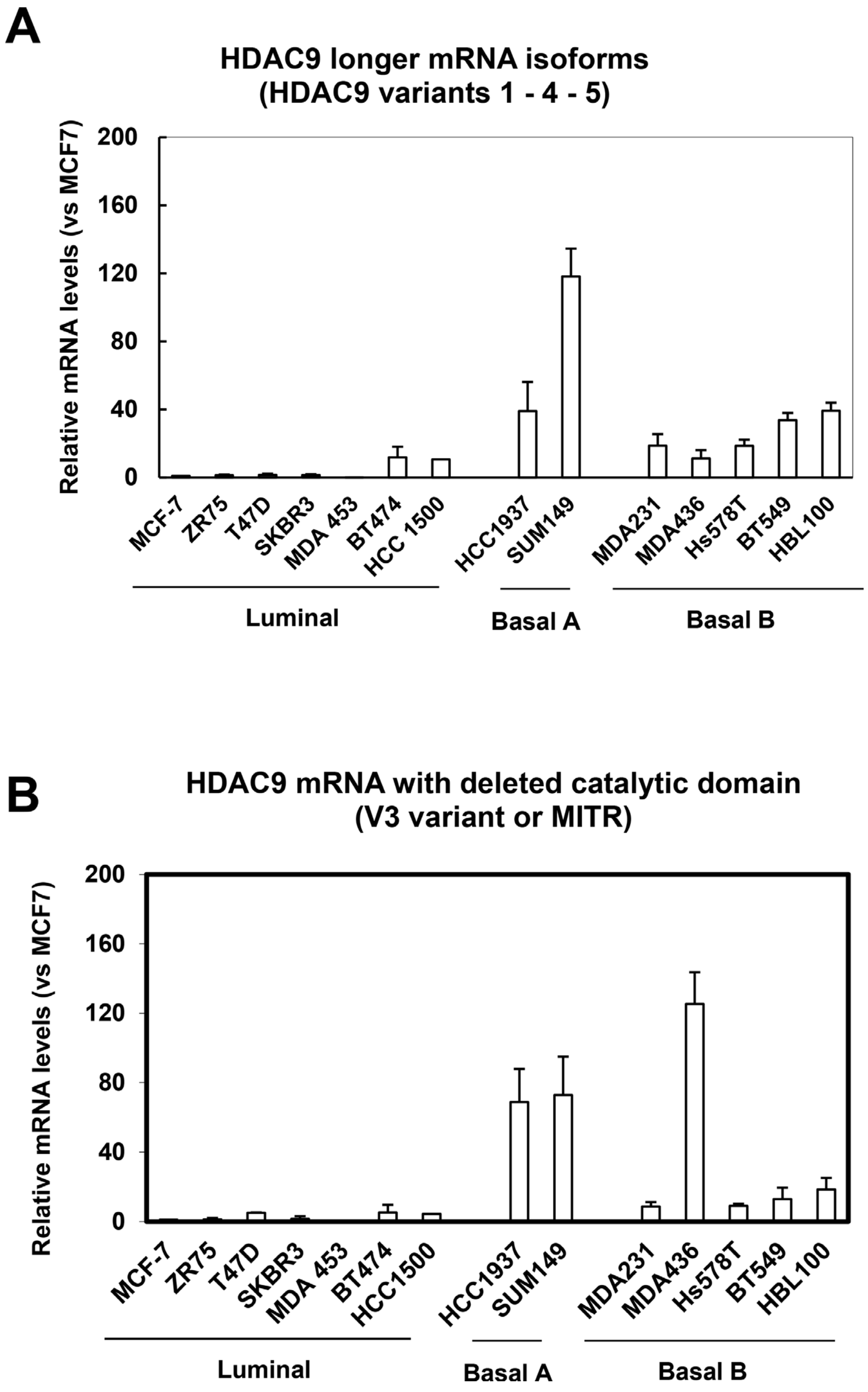

Supplementary Figure S2: Expression of HDAC9 mRNA isoforms in breast tumor cell lines. A. mRNA levels for long HDAC9 isoforms (V1-4-5) or **B.** HDAC9ΔCD isoform lacking the catalytic deacetylase domain (also known as V3 or MITR) were measured in fourteen breast tumor cell lines classified as luminal (n=7), basal A (n=2) and basal B (n=5). Results are expressed relative to the HDAC mRNA levels of the MCF7 cells and represent mean ± SD of 3 independent cell cultures.

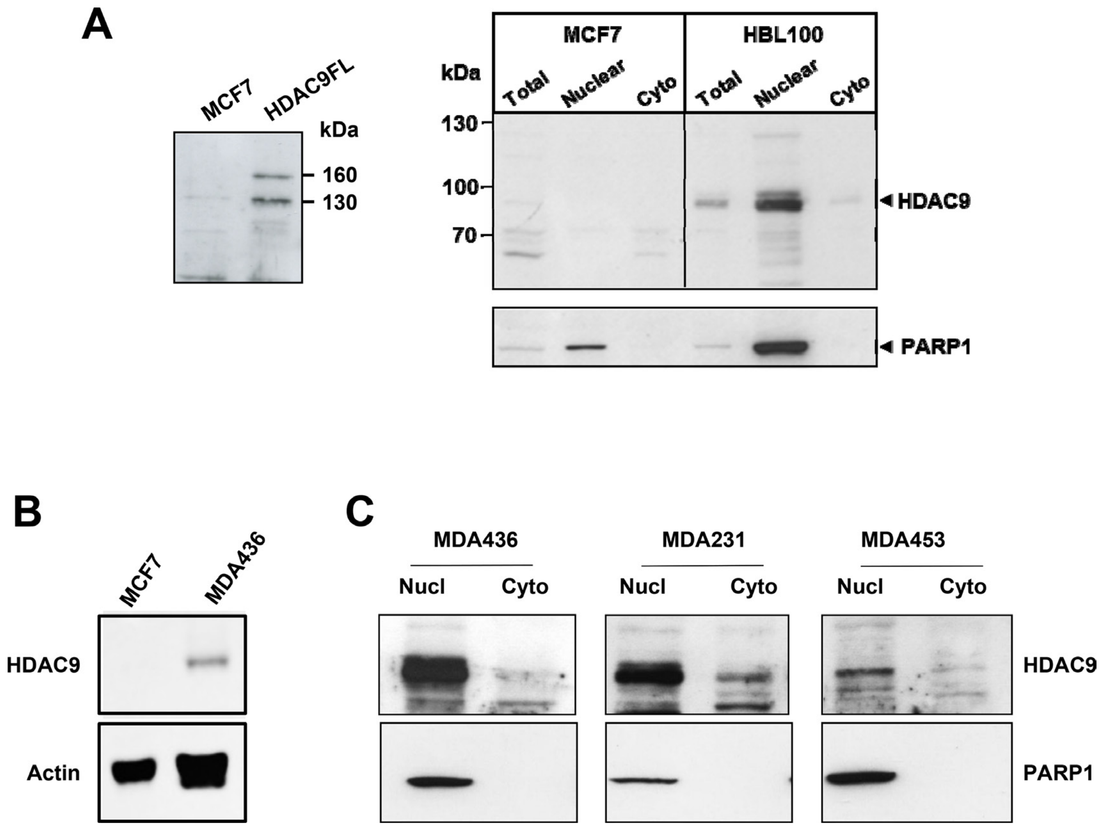

Supplementary Figure S3: Expression of the HDAC9 protein in human breast cancer cell lines. A. Protein were extracted from MCF7 cells transiently transfected with a plasmid containing full length HDAC9 (HDAC9FL) and analyzed by western-blot using anti-HDAC9 antibody. Non transfected MCF7 cells were used as control. Total, nuclear or cytoplasmic proteins were extracted from MCF7 or HBL100 cells and analyzed by western-blot using anti-HDAC9 antibody. PARP-1 was used as a loading control. This western-blot is representative of two independent experiments. **B.** Total proteins were extracted from MCF7 or MDA-MB436 cells and analyzed by western-blot using anti-HDAC9 antibody. Actin was used as a loading control. **C.** Same as in panel A in MDA-MB436, MDA-MB231 and MDA-MB453 breast cancer cell lines.

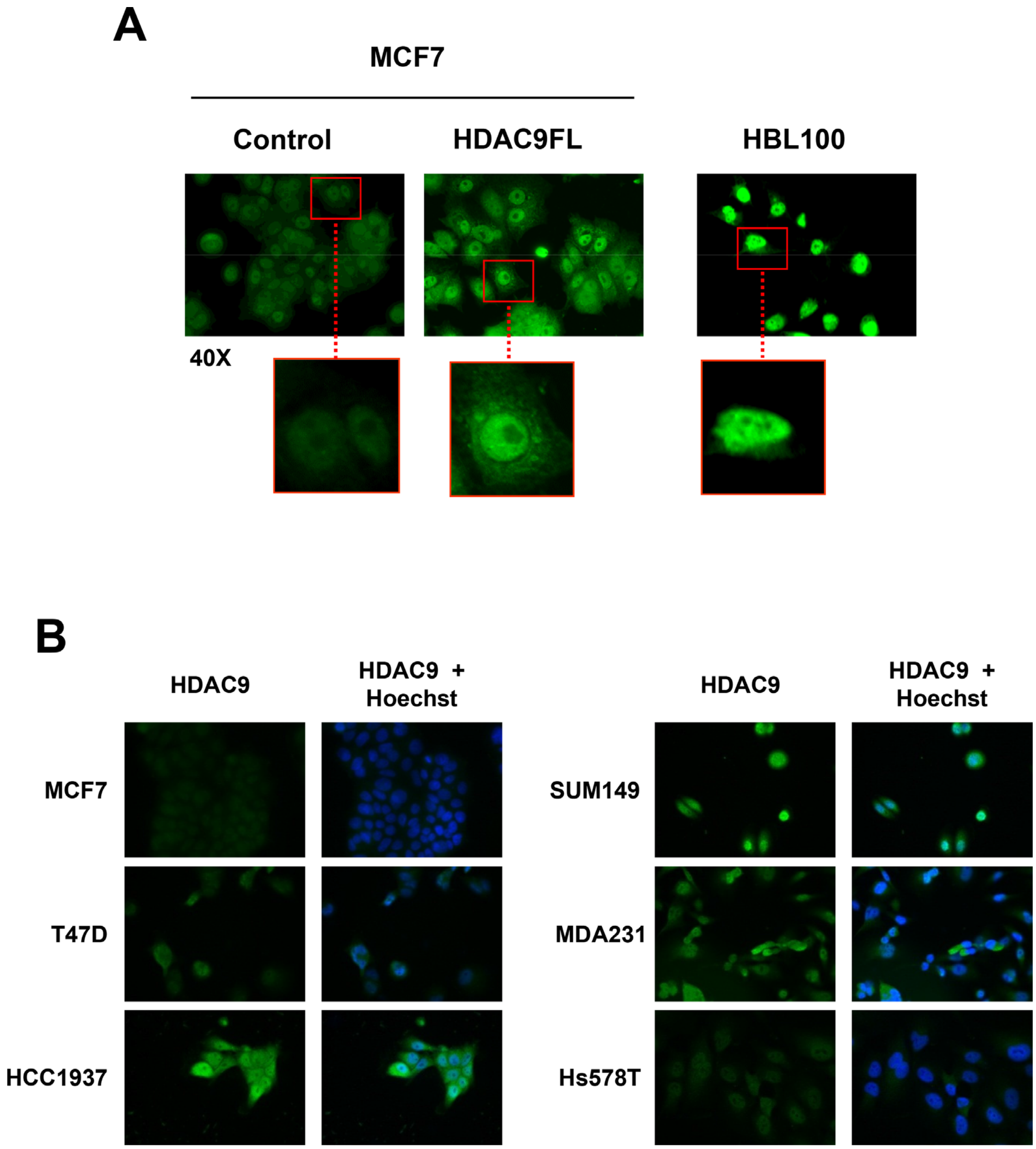

Supplementary Figure S4: Expression of the HDAC9 protein in human breast cancer cell lines. A. HDAC9 protein was analyzed by immunofluorescence in the MCF7 cells and in basal breast tumor cells HBL100. The small panels on the bottom represent individual cells. **B.** Immunofluorescence of endogenous HDAC9 protein in luminal and basal cells.

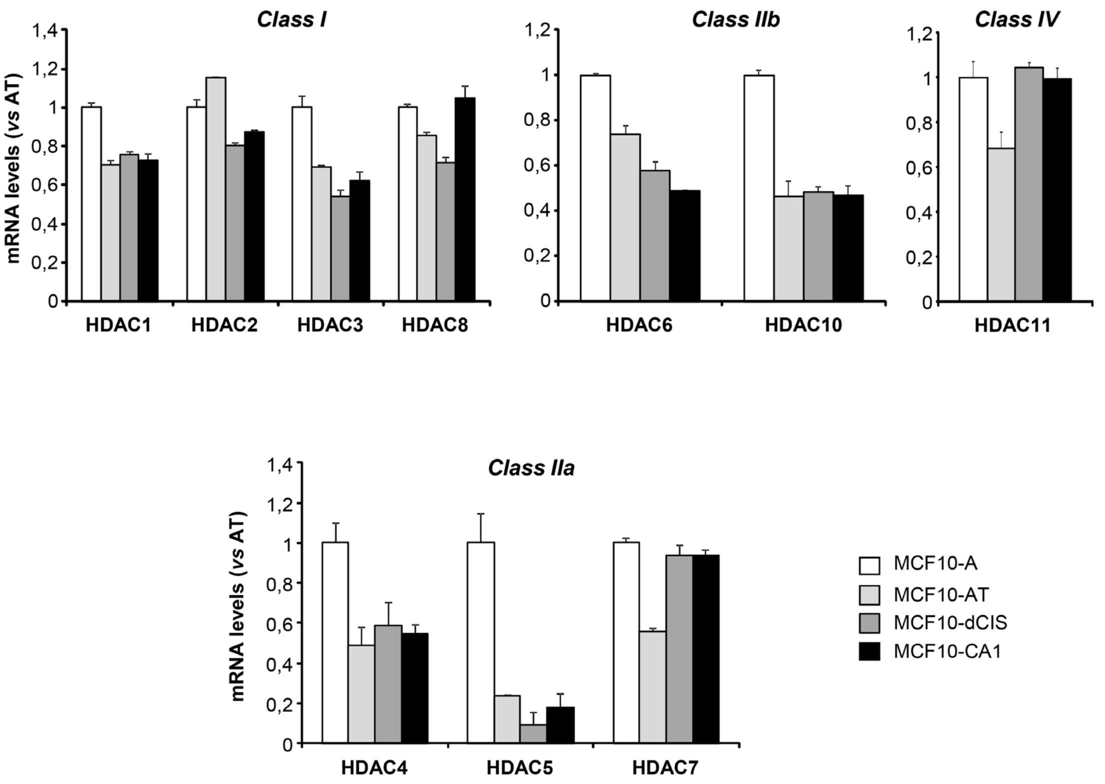

Supplementary Figure S5: HDAC expression in MCF10 mammary cell lines. MCF10 mammary cell lines were grown at confluence in FCS containing medium. The mRNA levels for class I, II and IV HDACs were measured using RT-qPCR. Results are expressed relative to the MCF10A cells used as reference. Results represent mean ± S.D. of 3 independent cell cultures.

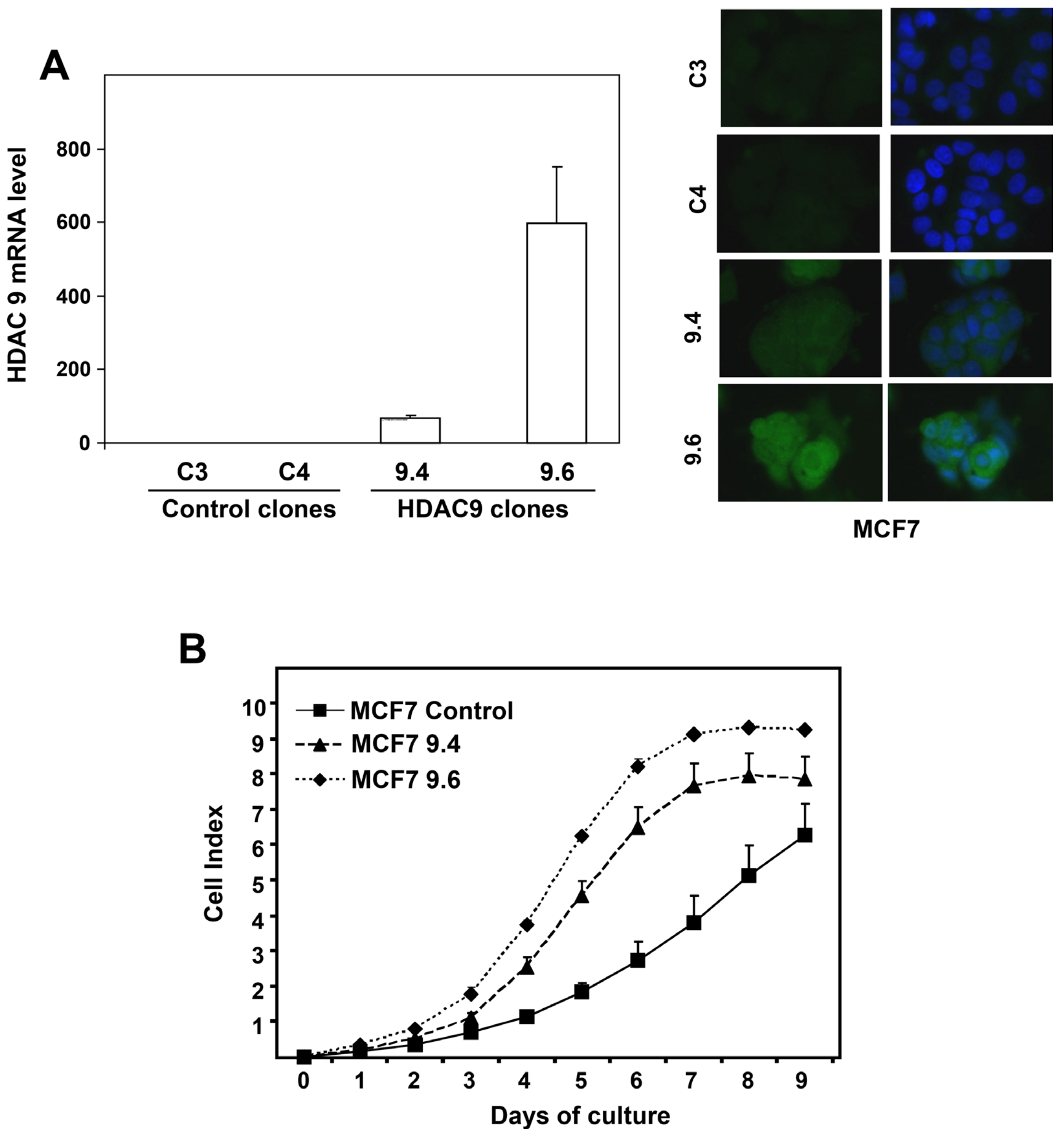

Supplementary Figure S6: Effects of HDAC9 overexpression on breast tumor cell proliferation. A. MCF7 cells were stably transfected using either control plasmid (Control clones C3 and C4) or full length HDAC9 plasmid (clones 9.4 and 9.6). HDAC9 mRNA levels were quantified using RT-qPCR. Results are expressed relative to the HDAC9 mRNA levels measured for the control MCF7 cells (left panel). MCF7 control clone and HDAC9 overexpressing clones (clones 9.4 and 9.6) were analyzed by immunofluorescence using anti-HDAC9 antibody and Hoechst labeling (right panel). **B.** Viable cells of MCF7 Control, 9.4 and 9.6 clones were monitored using the xCELLigence system every 24 hours during 9 days. Values are means ± S.D., n=3 independent experiments.

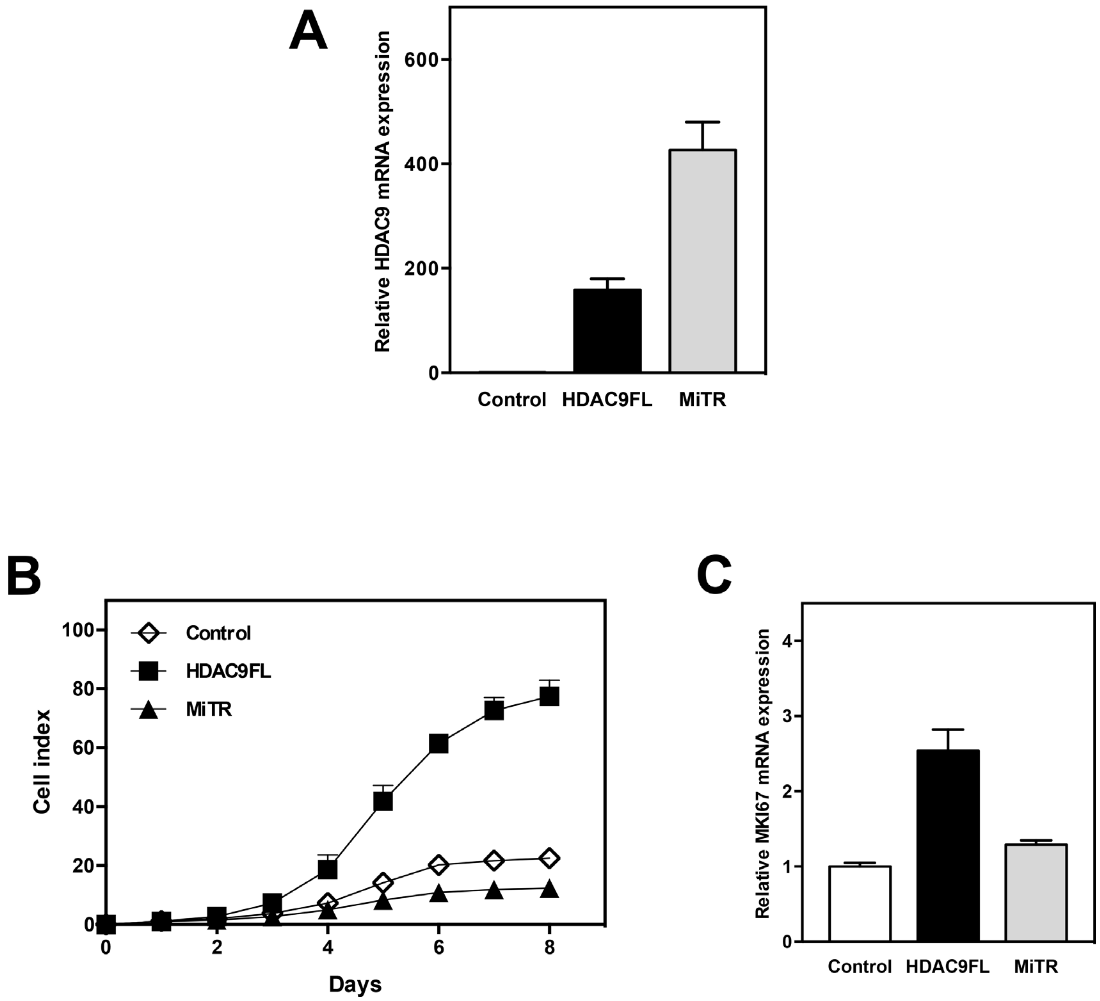

Supplementary Figure S7: Effect of HDAC9 MITR isoform on breast cancer cell proliferation. A. HDAC9 mRNA levels were quantified using RT-qPCR in MCF7 cells stably transfected using either an empty vector (Control) or a plasmid allowing the expression of full length HDAC9 (HDAC9FL) or the MITR isoform (MITR). Results are expressed in arbitrary units (AU) as mean ± S.D. of 6 independent cell cultures. **B.** Cell index corresponding to the number of MCF7-Control, MCF7-HDAC9FL and MCF7-MITR viable cells were monitored every 24 hours during 8 days using the xCELLigence system. Values are means ± S.D., n=3 independent experiments. **C.** Ki67 mRNA levels were quantified using RT-qPCR. Results represent fold change ± S.D. of 6 independent cell cultures *vs* levels in MCF7-Control cells after normalization to 28S mRNA.

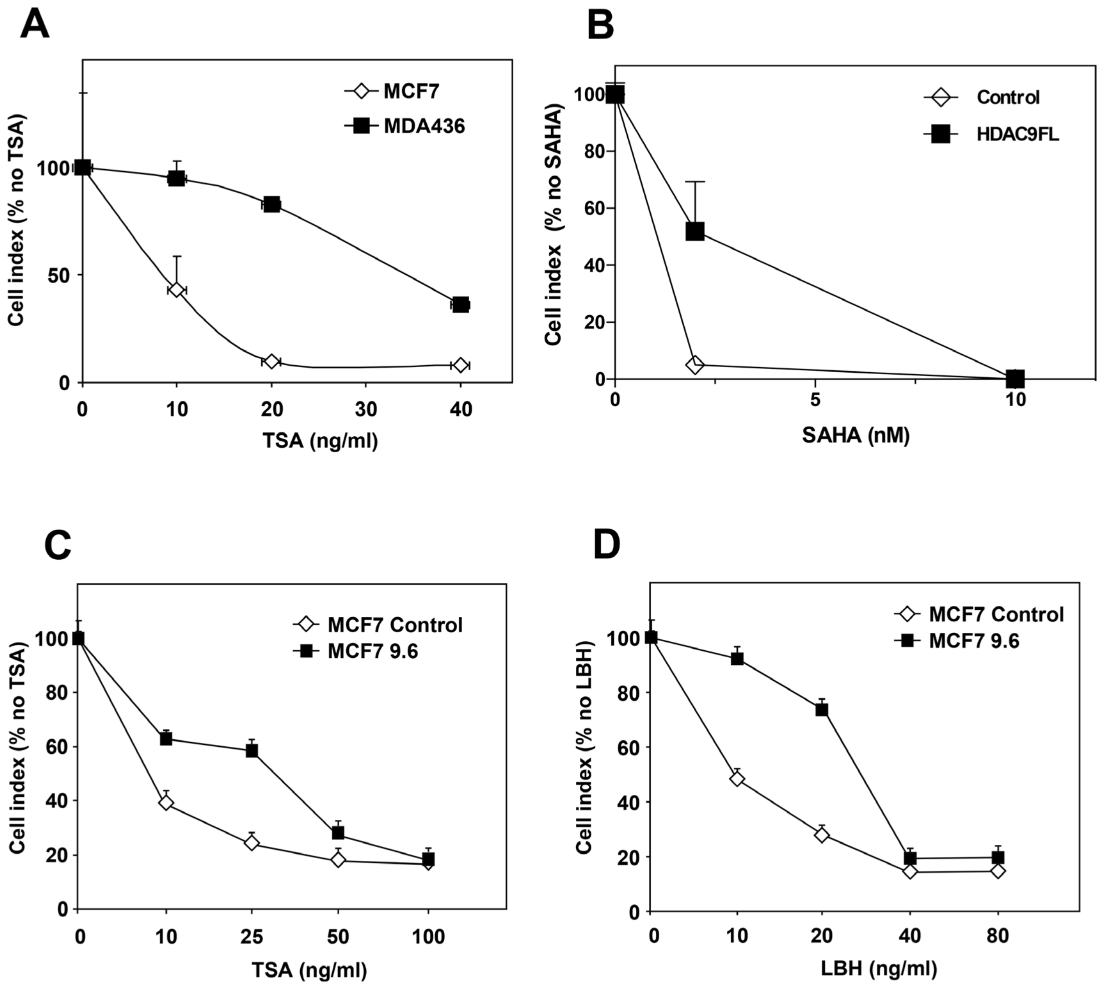

Supplementary Figure S8: Effects of HDAC9 overexpression on the response to HDIs. A. MCF7 and MDA-MB436 wild-type cells were treated with TSA at 10, 20, 30 or 40 ng/ml or solvent alone and viable cells were monitored using the xCELLigence system during 72 hours. Values are means ± S.D., normalized to solvent alone; n=3 independent experiments. **B.** MCF7-Control and MCF7-HDAC9FL cells were treated with increasing concentrations of SAHA (up to 10nM) or with solvent alone (Control) and viable cells were monitored using the xCELLigence system during 72 hours. **C.** MCF7 control clone and HDAC9 overexpressing clone 9.6 were treated with increased doses of TSA and viable cells were monitored using the xCELLigence system during 72 hours. Values are means ± S.D., normalized to solvent alone; n=3 independent experiments. **D.** Same as in panel C, with LBH 589 treatment.

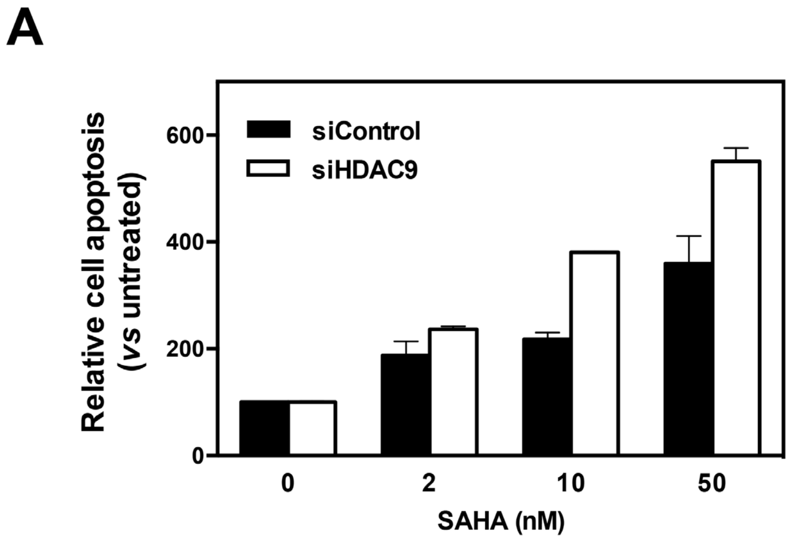

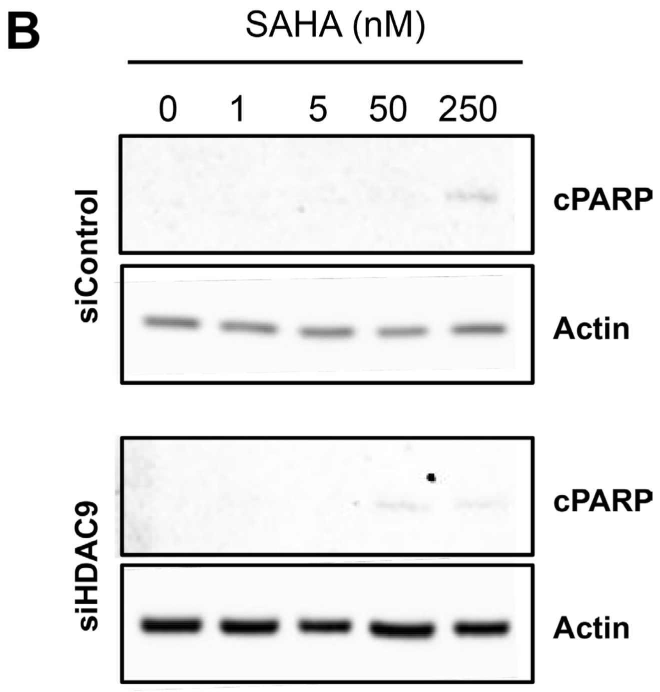

Supplementary Figure S9: Effects of HDAC9 on apoptosis in MDA-MB436 cells with HDAC9 knock-down. A. MDA436-siControl and MDA436-siHDAC9 cells were treated with increasing concentrations of SAHA (up to 50nM) or with solvent alone (0). The cell death detection kit (Roche) was used for the *in vitro* determination of cytoplasmic histone-associated-DNA-fragments (mono- and oligonucleosomes). Results are expressed as percent of untreated cells. **B.** Total proteins were extracted from MDA436-siControl and MDA436-siHDAC9 cells and analyzed by western-blot using an antibody against cleaved PARP (cPARP). Actin was used as a loading control.

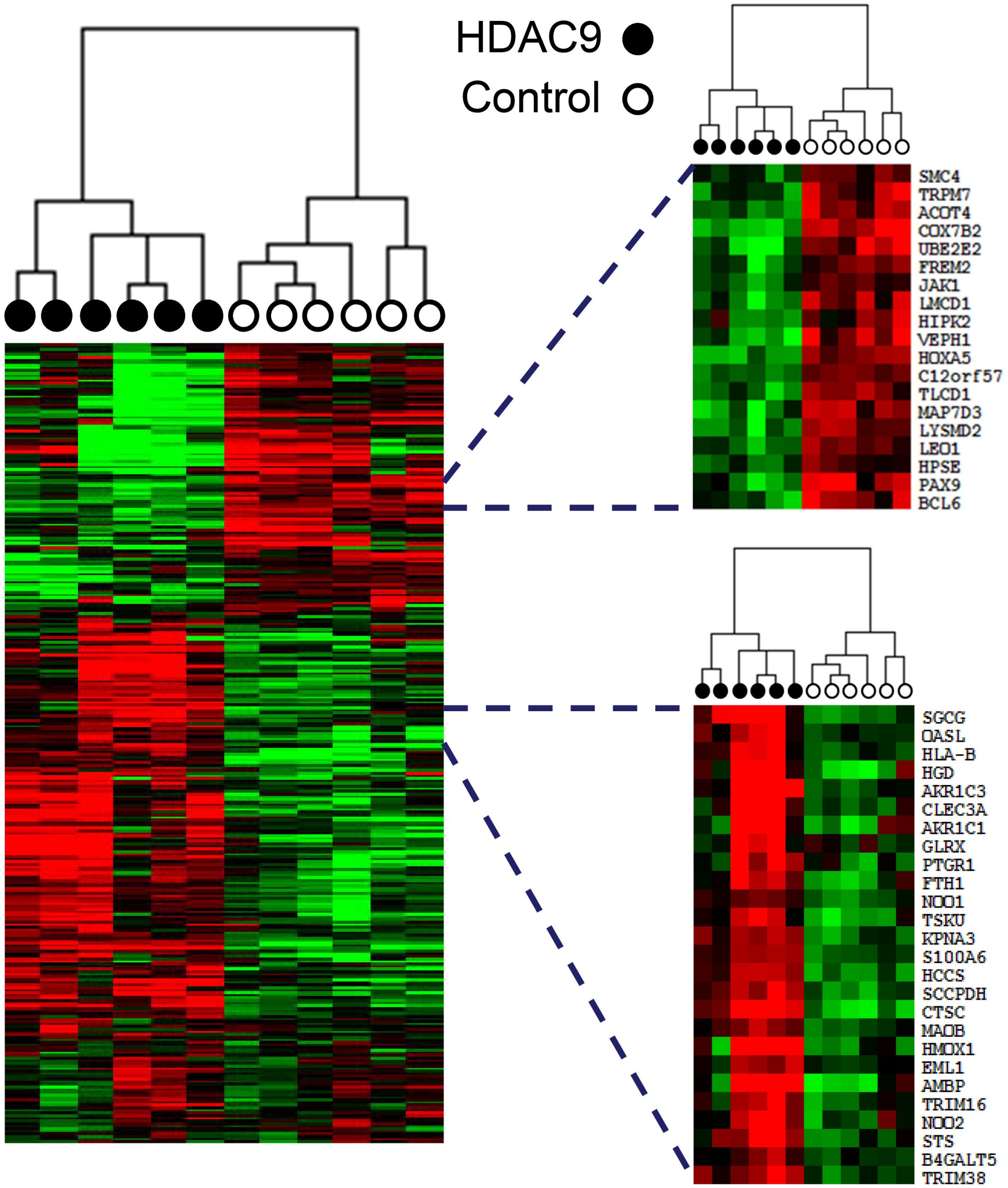

Supplementary Figure S10: Molecular signatures of MCF7-Control and MCF7-HDAC9FL cells. The molecular signatures of MCF7-Control and MCF7-HDAC9FL cells were visualized by hierarchical clustering on the 315 genes. The genes are arranged in rows and the samples are arranged in columns. In each cell categories, a tree represents relationship among samples whose branch lengths reflect the degree of similarity between the samples according to gene expression profile. In red, up-regulated genes; in green, down-regulated genes.

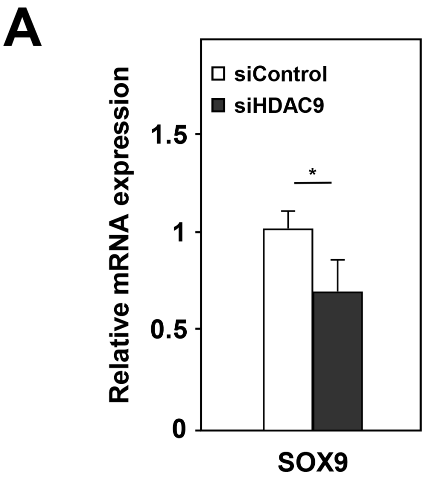

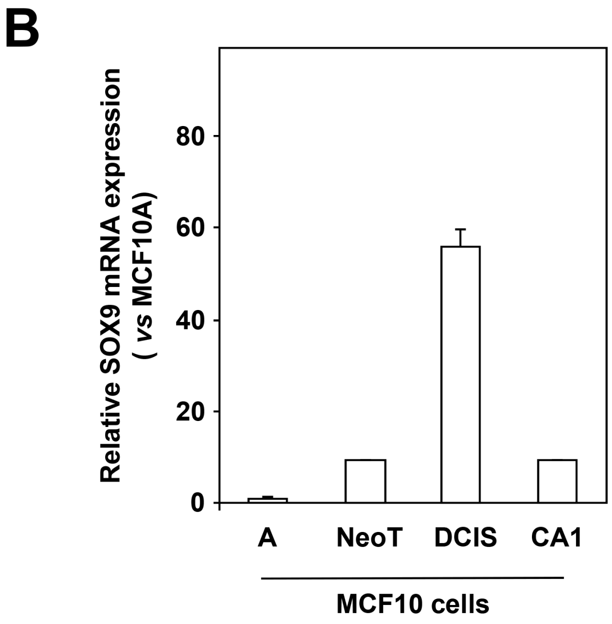

Supplementary Figure S11: Expression of SOX9 in breast cancer cell lines. A. SOX9 mRNA levels were measured by RT-qPCR in MDA-MB436 cells after silencing (siHDAC9) or not (siControl) of the *HDAC9* gene. Results are expressed relative to the SOX9 mRNA levels of the MDA-MB436 control cells and represent mean ± S.D., of 3 independent cell cultures *$p<0.01$. B. SOX9 mRNA levels were measured by RT-qPCR in MCF10 breast tumor cell lines. Results are expressed relative to the HDAC mRNA levels of the MCF10A cells and represent mean ± SD of 3 independent cell cultures.

Supplementary Table S1: List of the 195 up-regulated genes in control *vs* HDAC9FL MCF7 cells as determined by oligonucleotide microarray analysis

See Supplementary File 1

Supplementary Table S2: List of the 120 down-regulated genes in control *vs* HDAC9FL MCF7 cells as determined by oligonucleotide microarray analysis

See Supplementary File 1

Supplementary Table S3: Biological functions associated with the 315 genes differentially expressed in control vs HDAC9FL MCF7 cells

See Supplementary File 1

Supplementary Table S4: Primers sequences used for QPCR and RT-QPCR

See Supplementary File 1
