# Supplementary material for: Histone deacetylase 9 regulates breast cancer cell proliferation and the response to histone deacetylase inhibitors
Source: Oncotarget. 2016 Feb 22;7(15):19693–708. doi: 10.18632/oncotarget.7564 (PMC4991412; doi:10.18632/oncotarget.7564)
Supplement: Supplementary file 2 [file oncotarget-07-19693-s002.ppt]

## Slide 1
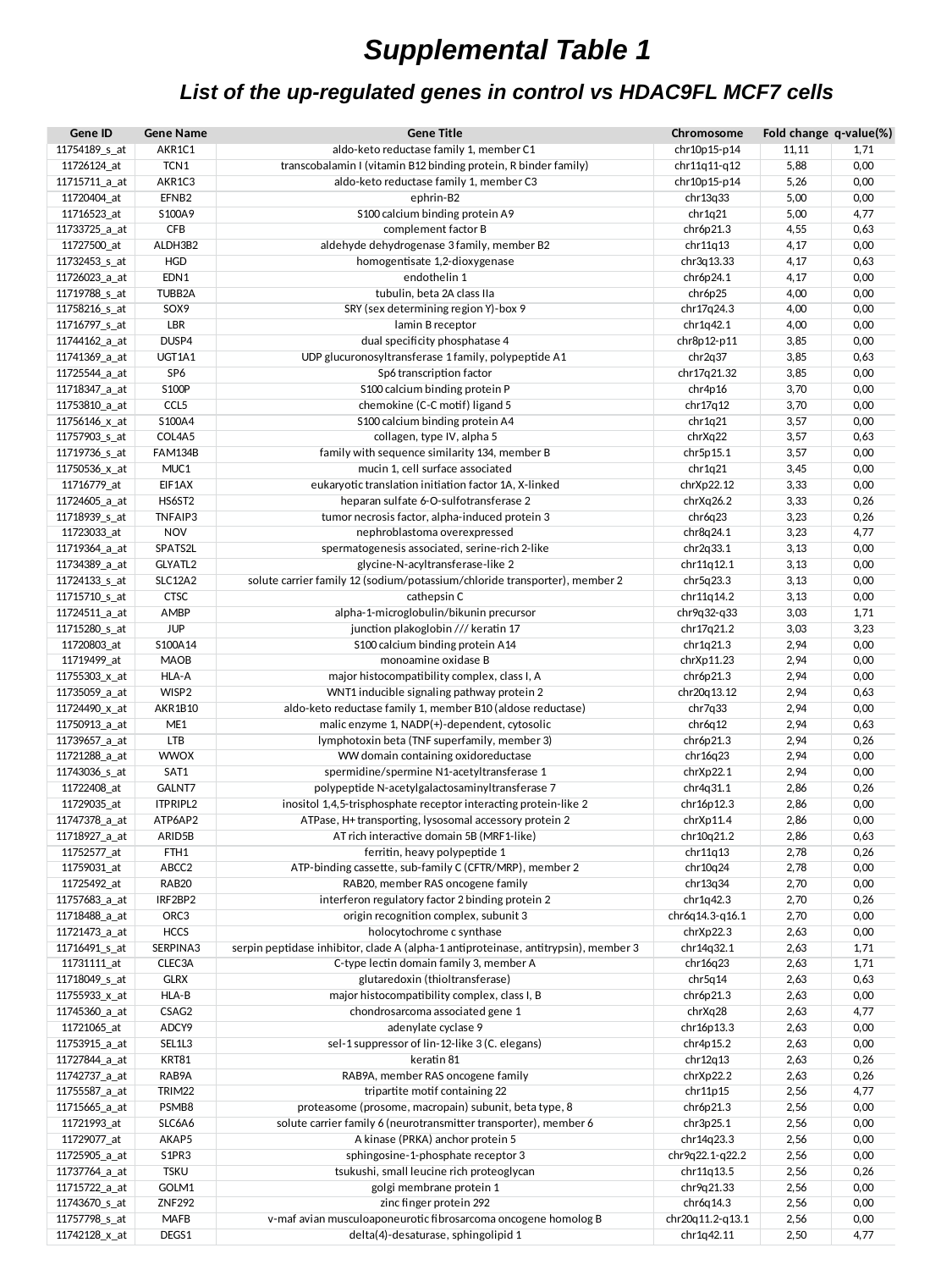

Supplemental Table 1
List of the up-regulated genes in control vs HDAC9FL MCF7 cells

## Slide 2
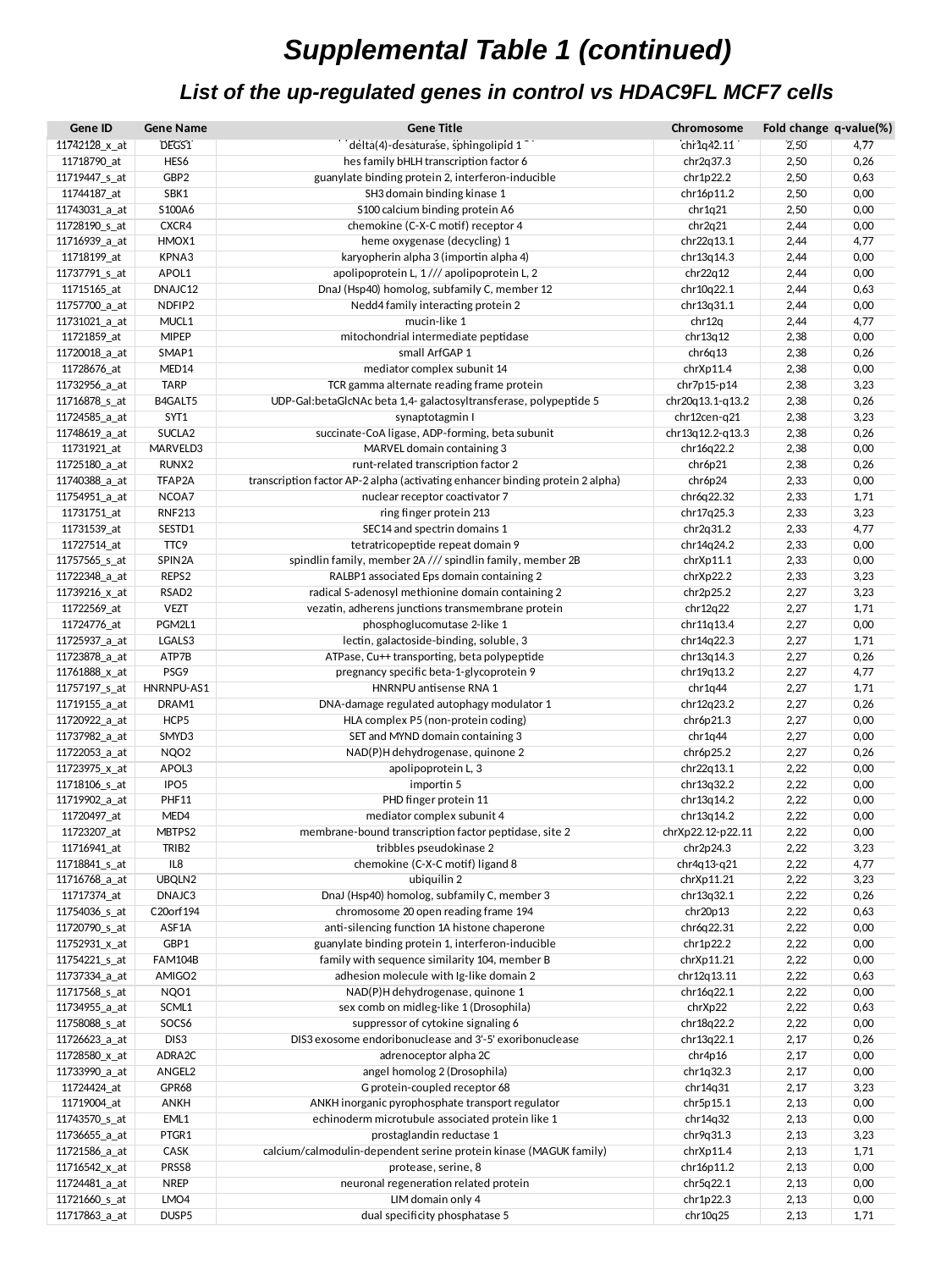

Supplemental Table 1 (continued)
List of the up-regulated genes in control vs HDAC9FL MCF7 cells

## Slide 3
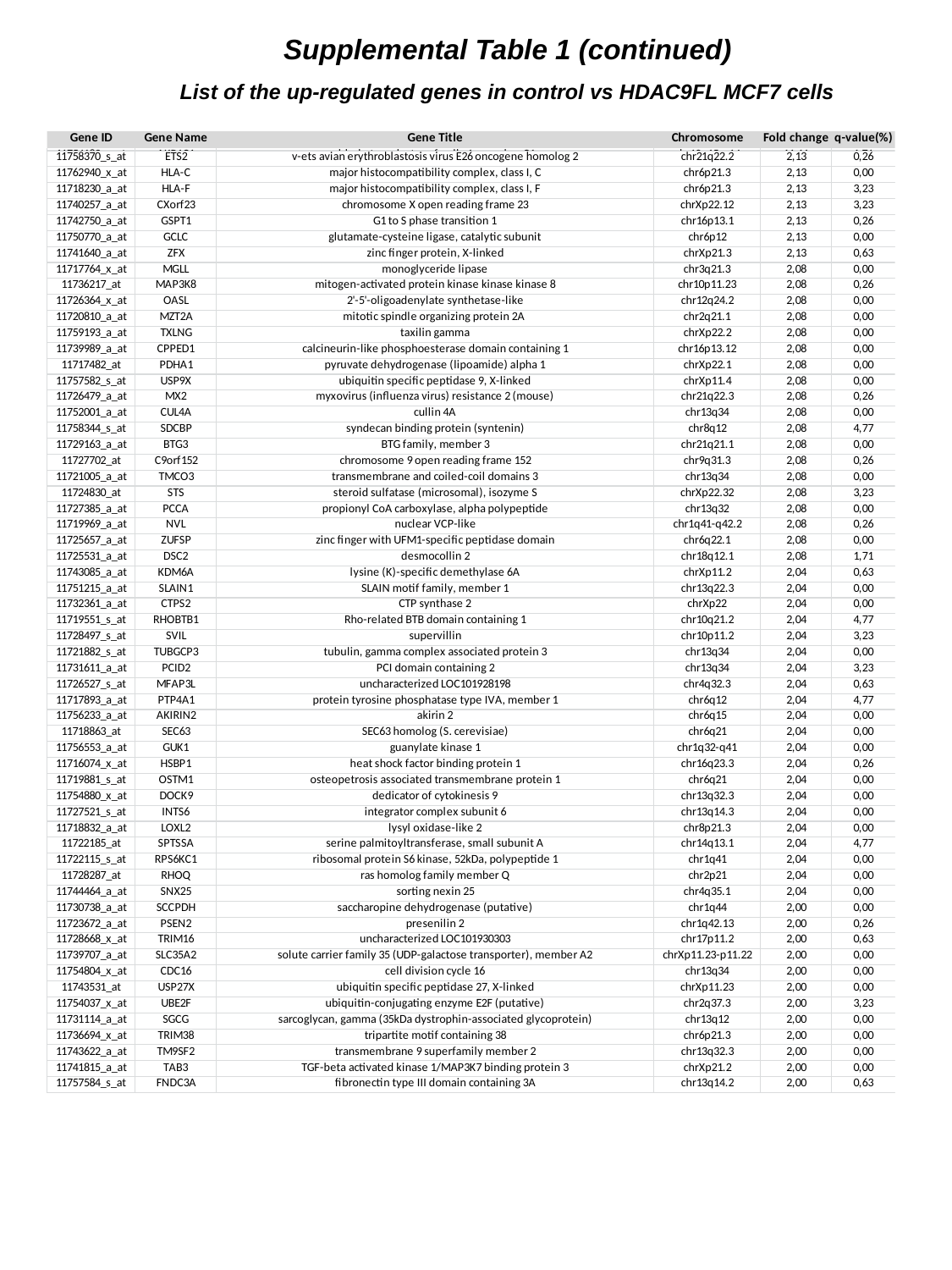

Supplemental Table 1 (continued)
List of the up-regulated genes in control vs HDAC9FL MCF7 cells

## Slide 4
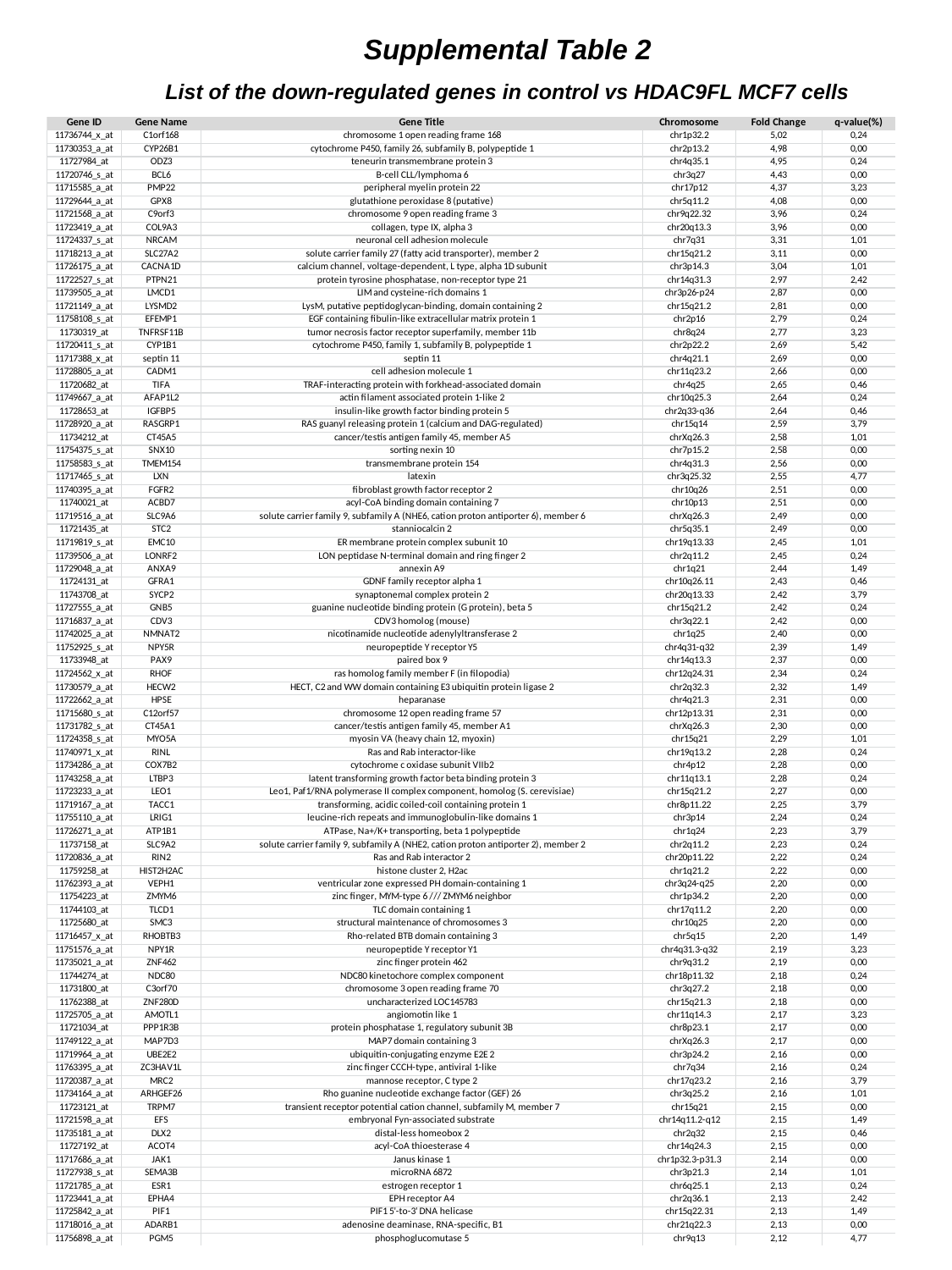

Supplemental Table 2
List of the down-regulated genes in control vs HDAC9FL MCF7 cells

## Slide 5
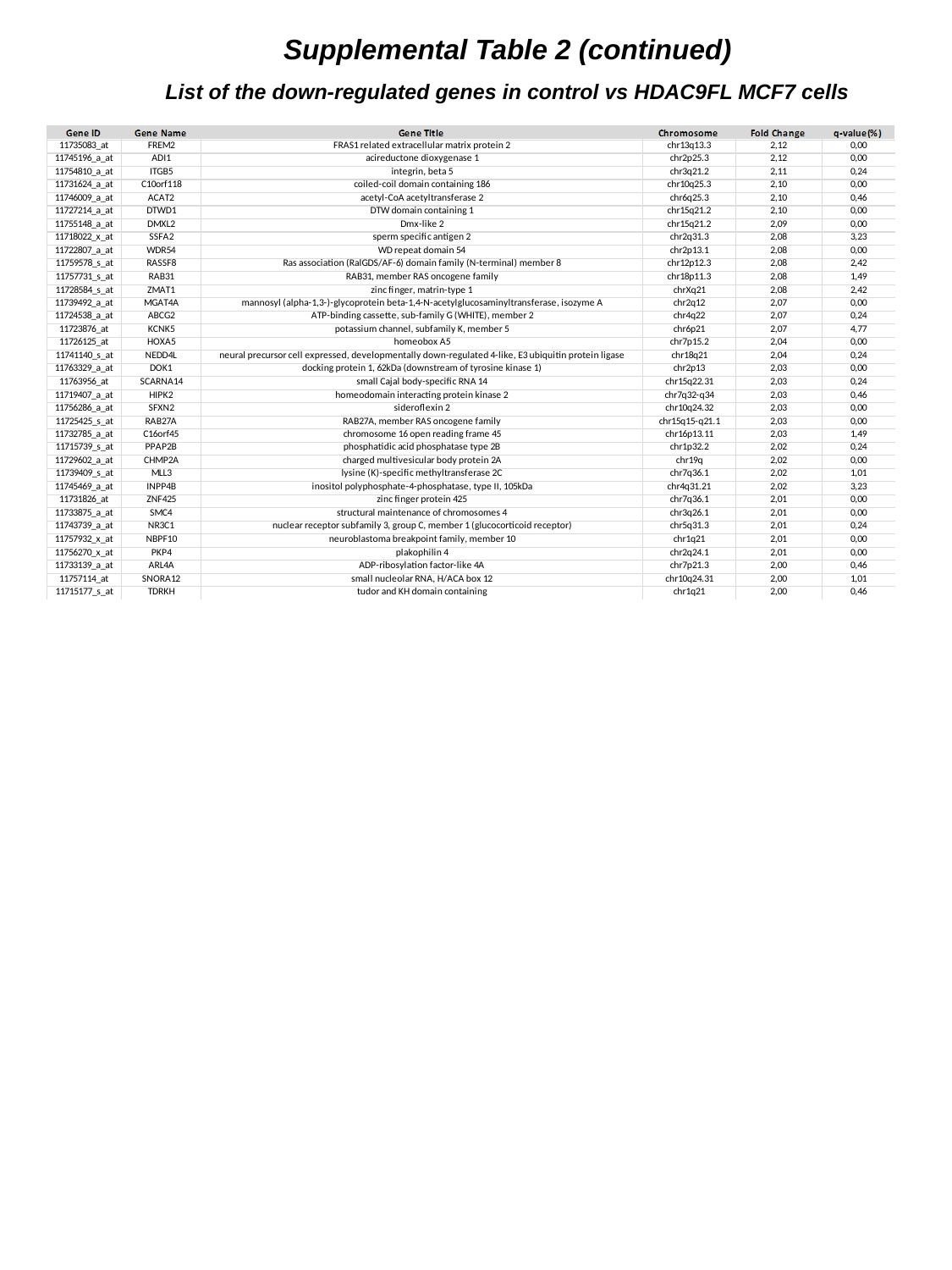

Supplemental Table 2 (continued)
List of the down-regulated genes in control vs HDAC9FL MCF7 cells

## Slide 6
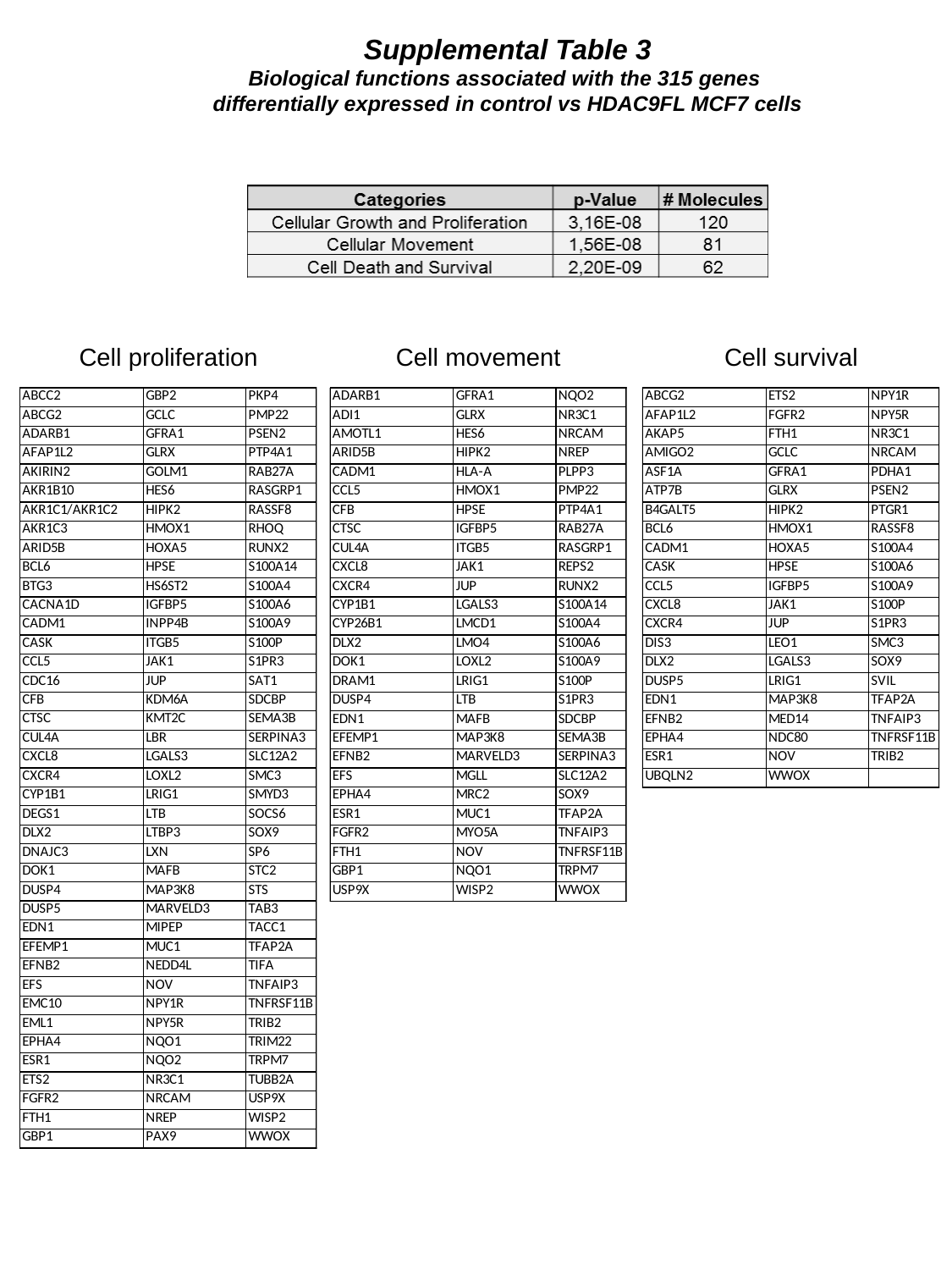

Supplemental Table 3
Biological functions associated with the 315 genes
differentially expressed in control vs HDAC9FL MCF7 cells
Cell proliferation
Cell movement
Cell survival
| Categories | p-Value | # Molecules |
| --- | --- | --- |
| Cellular Growth and Proliferation | 3,16E-08 | 120 |
| Cellular Movement | 1,56E-08 | 81 |
| Cell Death and Survival | 2,20E-09 | 62 |

## Slide 7
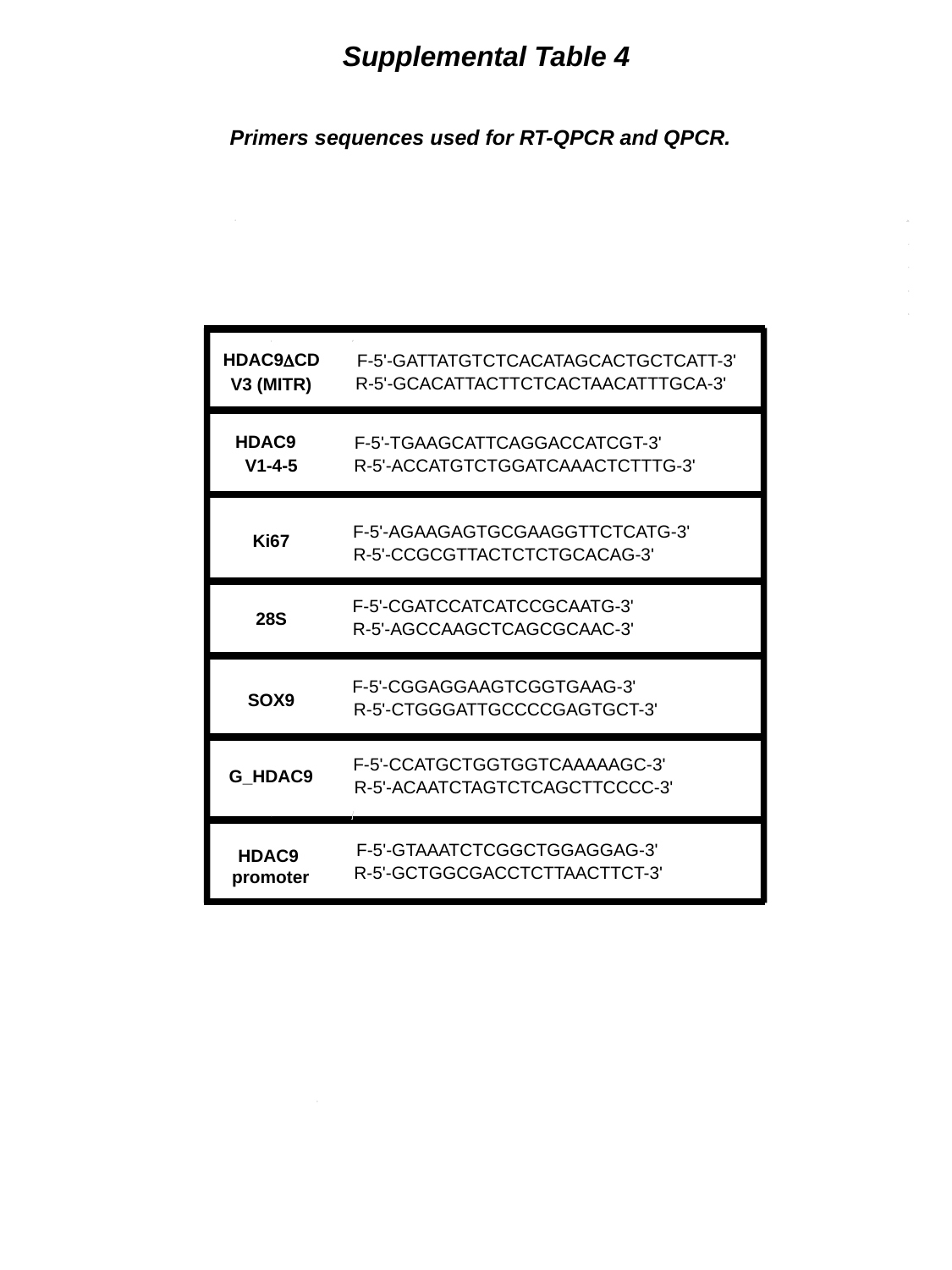

Supplemental Table 4
Primers sequences used for RT-QPCR and QPCR.
HDAC9ΔCD
V3 (MITR)
F-5'-GATTATGTCTCACATAGCACTGCTCATT-3'
R-5'-GCACATTACTTCTCACTAACATTTGCA-3'
HDAC9
V1-4-5
F-5'-TGAAGCATTCAGGACCATCGT-3'
R-5'-ACCATGTCTGGATCAAACTCTTTG-3'
F-5'-AGAAGAGTGCGAAGGTTCTCATG-3'
Ki67
R-5'-CCGCGTTACTCTCTGCACAG-3'
F-5'-CGATCCATCATCCGCAATG-3'
28S
R-5'-AGCCAAGCTCAGCGCAAC-3'
F-5'-CGGAGGAAGTCGGTGAAG-3'
SOX9
R-5'-CTGGGATTGCCCCGAGTGCT-3'
F-5'-CCATGCTGGTGGTCAAAAAGC-3'
G_HDAC9
R-5'-ACAATCTAGTCTCAGCTTCCCC-3'
F-5'-GTAAATCTCGGCTGGAGGAG-3'
HDAC9
promoter
R-5'-GCTGGCGACCTCTTAACTTCT-3'
